# Secure image exchange over cloud networks with neural key exchange protocol

**DOI:** 10.1038/s41598-026-66952-w

**Published:** 2026-08-25

**Authors:** Wassim Alexan, Youstina Megalli, Maran Malak, Salma Khaled, Maggie Mashaly

**Affiliations:** 1 Communications Department, Faculty of Information Engineering and Technology, German University in Cairo (GUC), New Cairo, Egypt; 2 Computer Science Department, Faculty of Media Engineering and Technology, German University in Cairo (GUC), New Cairo, Egypt; 3 Networking Department, Faculty of Information Engineering and Technology, German University in Cairo (GUC), New Cairo, Egypt

**Keywords:** Cellular automata, Image encryption, Mersenne twister, Tree parity machines, NIST analysis, S-box, Computational biology and bioinformatics, Engineering, Mathematics and computing

## Abstract

In this article, a novel image encryption algorithm is proposed for the secure transmission and storage of images over the cloud. The robustness of Tree Parity Machines (TPMs) is utilized in conjunction with DNA coding, Mersenne Twister-based DNA sequence generation, SHA-512 seeded Rule 30 Cellular Automata (CA), and S-box construction to achieve comprehensive multi-layered image encryption. The encryption process is initiated through the use of TPMs, which are employed to facilitate secure key exchange among users communicating over the cloud. The weight vectors of the TPMs are utilized as an initial shared symmetric key, and through iterative weight synchronization, the final weights are evolved into the actual encryption keys. DNA coding is then applied as the first layer of encryption. The corresponding DNA sequences are generated using the Mersenne Twister, which is employed as a Pseudo-Random Number Generator (PRNG). Subsequently, the SHA-512 hashing function is used to generate a seed for Rule 30 Cellular Automata, which is further utilized to construct another PRNG. This newly generated PRNG is applied as an additional key in an XOR operation with the image data, thereby providing an extra layer of security. The same CA-based PRNG is also used to construct an S-box, which is applied in the final encryption stage. Excellent results in terms of security, resistance to attacks, and computational efficiency are demonstrated through performance evaluation. The algorithm is shown to possess strong potential for secure and efficient real-time multimedia transmission and storage over the cloud. A significant advancement in secure cloud-based image communication is offered through the integration of TPMs, DNA coding, and CA in this unique encryption scheme. Moreover, quantitative evaluation further confirms the robustness of the proposed scheme, achieving encrypted image entropy of 7.99904, a high NPCR of *99.60897%* and a UACI of *32.26675%*, reflecting strong sensitivity to plain text variations, while maintaining an MSE of 10141.88 and PSNR of 8.11 dB.

## Introduction

The ubiquity of cloud-backed storage and content distribution has been accompanied by a transformation in how high-volume multimedia assets, particularly images, are produced, shared, and consumed across geographically dispersed and heterogeneous endpoints^1,2^. Applications ranging from telemedicine and medical imaging archives to intelligent transportation systems, remote sensing, and surveillance are characterized by the continuous exchange of sensitive visual data over semi-trusted or untrusted networks^3,4^. Through this data movement, the attack surface is amplified and the confidentiality, integrity, and availability of images are exposed to risks such as eavesdropping, ciphertext manipulation, key compromise, metadata leakage, and traffic analysis^5^. Although conventional block ciphers and stream ciphers are supported by well-studied security assumptions, the deployment of such ciphers for real-time image services over cloud environments can be constrained by: (i) overhead introduced by key distribution and rotation across multi-tenant, ephemeral, or serverless deployments^6^; (ii) fragility of modes of operation when ciphertext malleability or format constraints are present^7^; (iii) performance variability driven by large payloads, data-parallel pipelines, and device heterogeneity^8,9^; and (iv) diffusion and confusion that may be insufficient against image-specific statistics, for example large spatial correlations and nonuniform histograms, which are commonly exploited by cryptanalysis targeting visual data^10^.

In this work, a multi-layer image encryption framework is proposed in which neural synchronization-based key establishment is combined with multiple statistically orthogonal diffusion and confusion mechanisms tailored to image characteristics. Specifically, Tree Parity Machines (TPMs) are utilized to realize an interactive key agreement protocol over a public channel. The emergent synchronized weight vectors are used as high-entropy, session-specific symmetric material without reliance on pre-shared secrets or public-key certificates^11,12^. From these TPM-derived secrets, seeds for distinct pseudo-random number generators (PRNGs) and substitution structures are deterministically derived and are then used to drive complementary encryption layers^13^: (i) bitwise masking with PRNG key-streams; (ii) content-preserving DNA coding disturb local intensity statistics while invertibility is maintained; and (iii) S-box-based nonlinear substitution that is constructed and quality-gated via cryptographic criteria, including nonlinearity (NL), strict avalanche criterion (SAC), bit independence criterion (BIC), linear approximation probability (LAP), and differential approximation probability (DAP)^14–17^. Diversification of entropy sources is achieved by employing a Mersenne Twister (MT) generator and a SHA-512 seeded Rule-30 elementary cellular automaton (CA) so that structural biases are reduced and resistance against known-plaintext and chosen-plaintext attacks is strengthened^18,19^.

At a high level, the proposed system is composed of:

- Key establishment: a TPM two-party synchronization protocol by which two final weight vectors are yielded. By applying SHA-512 over these vectors, seeds that parameterize the subsequent cryptographic primitives are generated.
- Diffusion and confusion: three encryption layers, namely (L1) XOR masking and S-box substitution driven by MT; (L2) DNA mapping and DNA arithmetic, for example addition or subtraction, by which spatial statistics are decorrelated at the symbol level; and (L3) CA-driven XOR masking followed by an independently generated S-box substitution. Through these layers, diffusion, nonlinearity, and avalanche effects are jointly intensified.
- Adaptive S-box synthesis: an iterative synthesis-and-test process by which S-boxes are accepted only when a configurable proximity threshold to idealized cryptographic indicators is met, so that weak instantiations are mitigated and robust substitution behavior is ensured.

Adherence to the design principle of defense in depth is followed by this architecture, and orthogonal security properties are contributed by each layer^20^. Static key provisioning is eliminated by TPM synchronization; a long period and high-dimensional equidistribution are provided by MT; computationally efficient key-streams with favorable randomness indicators are produced by Rule-30 CA^21^; structured yet invertible symbol transformations resistant to histogram and correlation-based analysis are introduced by DNA operations; and increased nonlinearity that complicates linear and differential cryptanalysis is provided by vetted S-boxes. The overall pipeline is engineered to remain lightweight and parallelizable so that alignment with real-time constraints common in cloud-based image services is achieved^9^.

The threat model in this work is defined for a cloud/network environment in which the communication channel is public and the cloud is semi-trusted. It is assumed that an adversary can (i) eavesdrop on all transmitted data, including TPM synchronization messages (shared input vectors and exchanged output bits) and ciphertext images, and (ii) store and analyze observed traffic offline. Known-plaintext and chosen-plaintext settings are further considered, in which plaintext-ciphertext pairs may be obtained or encryptions of chosen images may be requested within a session. The security objective is confidentiality of the image and the computational infeasibility of recovering session keys or distinguishing ciphertext structure from random.

For active attacks, it is assumed that TPM synchronization messages are authenticated (e.g., via a MAC, certificates, or a secure side channel). Without authentication, independent synchronized weights could be established by a man-in-the-middle (MITM) adversary with each party, similar to unauthenticated key agreement protocols. Finally, confidentiality is prioritized by the proposed scheme and an explicit integrity mechanism is not included; therefore, resistance to chosen-ciphertext/decryption-oracle attacks is not claimed unless an authentication layer (e.g., Encrypt-then-MAC or AEAD) is combined with the scheme.

Although the proposed scheme is applicable to cloud networks, the cloud environment introduces threats beyond a traditional point-to-point channel. In particular, multi-tenancy and shared physical resources may enable cross-tenant leakage (e.g., via side channels), virtualization-layer compromise (VM/container escape or malicious hypervisors) may expose data in memory, and insecure cloud APIs/IAM misconfigurations (e.g., overly permissive roles, exposed object storage, snapshot/backup access) may lead to unauthorized disclosure or tampering^22^. The proposed method primarily mitigates confidentiality risks for data in transit and at rest by ensuring that images stored or routed through a semi-trusted cloud remain unintelligible without the session keys derived from authenticated TPM synchronization. However, protection against endpoint compromise, malicious host/hypervisor memory inspection, or control-plane/API takeover is outside the cipher’s scope and should be addressed with complementary cloud controls (e.g., strong IAM and audit policies, secure key management/HSM/KMS, TLS, and optionally TEEs), and integrity should be ensured by deploying AEAD or Encrypt-then-MAC alongside the encryption pipeline^23^.

The main technical contributions of this manuscript are:

1. Neural key agreement for images: a practical integration of Tree Parity Machine synchronization is presented as a certificate-free, session-oriented key exchange for cloud image encryption, and reliance on pre-distributed symmetric keys or heavyweight public-key infrastructures in multi-tenant settings is eliminated.
2. Dual-PRNG entropy diversification: a deterministic derivation of two independent key-stream sources from TPM outputs, namely Mersenne Twister and SHA-512 seeded Rule-30 CA, is achieved so that structural correlation is reduced and resistance against distinguishing attacks and PRNG-specific weaknesses is enhanced. The synchronized weight vectors $W_1$ and $W_2$ are never transmitted. Thus, attacks such as known-plaintext attacks (KPA) become infeasible.
3. DNA-coded confusion and diffusion: a reversible DNA coding layer that operates at the nucleotide-symbol level, with well-defined arithmetic and logical operations, is employed so that adjacent pixels are decorrelated and image histograms are flattened while exact invertibility is preserved.
4. Quality-gated S-box construction: a seed-dependent S-box generation mechanism driven by MT and CA seeds is used, and acceptance is performed only when composite cryptographic metrics, including nonlinearity, SAC, BIC, LAP, and DAP, meet configurable thresholds. By this procedure, high-quality nonlinear substitution layers are yielded.
5. End-to-end, three-layer cipher design: a cohesive pipeline is constructed by which XOR masking, S-box substitution, and DNA arithmetic are combined in an alternating sequence, namely PRNG to S-box to DNA to CA-PRNG to S-box, so that avalanche behavior is strengthened and differential and linear cryptanalysis is complicated.
6. Comprehensive security evaluation: an extensive suite of empirical tests, including histogram uniformity, information entropy, inter-pixel correlation, MSE and PSNR, MAE, NPCR and UACI, key-space estimation, execution time, and NIST SP 800-22 randomness tests, is conducted. Through these evaluations, strong confusion and diffusion, resistance to statistical and differential attacks, and competitive runtimes suitable for real-time or near real-time cloud operation are demonstrated.
7. Cloud suitability: an implementation strategy and parameterization compatible with cloud deployments, where session keys must be frequently refreshed and endpoints are usually heterogeneous, are provided so that scalable and secure multimedia storage and transmission are supported.

The key novelty beyond integration of various value-adding constructs is described as follows. Although TPM synchronization, DNA operations, Rule-30 cellular automata, and the Mersenne Twister are individually established, a synchronization-conditioned key/key-stream construction is introduced in which these components are coupled in a manner that measurably alters security and robustness behavior. Specifically, seed material is derived from the final synchronized TPM weight vectors, and cryptographic hashing is applied to obtain fixed-length, high-entropy seeds. Two independently generated pseudo-random streams (Rule-30 CA-derived and MT-derived) are then driven by these seeds, assigned distinct roles in the cipher pipeline (e.g., one primarily for confusion/substitution control and the other for diffusion/masking), and subsequently mixed. Through this separation-and-mixing design, reliance on any single generator is reduced, and the effective key material is bound to the synchronized dynamics of the communicating parties rather than to a static, externally chosen key. It is important to note that TPM is used as a lightweight, interactive key agreement supported mainly by empirical/parameter-based analyses; it is not claimed to provide DH/ECC-style proof-based AKE security; deployments needing provable AKE can swap in (EC)DH without changing the rest of the pipeline.

The quantifiable distinctions of this research work are then provided as follows. Measurable gains produced by this mechanism are validated against representative baselines that use only TPM-derived keys, only CA-based key-streams, or DNA operations without synchronization-bound seeding, as reported in the performance evaluation section (“Performance analysis and evaluation” section). Information entropy closer to the ideal 8 bits/pixel, lower adjacent-pixel correlation in horizontal/vertical/diagonal directions, NPCR/UACI values consistently near ideal under one-pixel/plaintext perturbations, and stable NIST SP 800-22 pass rates across multiple images and runs are obtained, while competitive runtime is maintained. These results indicate that benefits beyond a simple concatenation of known modules are provided by the proposed synchronization-bound stream composition.

The remainder of this article is organized as follows. “Preliminary ideas” section provides a summary of the preliminaries. “Methodology of the proposed TPM image encryption scheme” section provides details of the proposed methodology. “Performance analysis and evaluation” section presents the security analysis and numerical results. Finally, Conclusions section provides the conclusions and outlines directions for future work. Table 1 provides the various acronyms utilized in this article.

**Table 1:** List of acronyms utilized in this article.

Acronym	Definition	Acronym	Definition
AEAD	Authenticated Encryption with Associated Data	AES	Advanced Encryption Standard
AKE	Authenticated Key Exchange	API	Application Programming Interface
BIC	Bit Independence Criterion	BPSK	Binary Phase Shift Keying
CA	Cellular Automaton/Cellular Automata	PCC	Pixel Cross Correlation Coefficient
CPU	Central Processing Unit	DAP	Differential Approximation Probability
DFT	Discrete Fourier Transform	DNA	Deoxyribonucleic Acid
(EC)DH	(Elliptic-Curve) Diffie–Hellman	EKB	Egyptian Knowledge Bank
ESM	Exponential Sine Map (as in 2D-ESM)	FFT	Fast Fourier Transform
FPGA	Field-Programmable Gate Array	GA	Genetic Algorithm
HMAC	Hash-based Message Authentication Code	HSM	Hardware Security Module
IAM	Identity and Access Management	IoT	Internet of Things
KMS	Key Management Service	LA	Left Almost (left almost semigroup)
LAP	Linear Approximation Probability	LDPC	Low-Density Parity-Check (code)
LFSR	Linear Feedback Shift Register	MAC	Message Authentication Code
MAE	Mean Absolute Error	MITM	Man-in-the-Middle (attack)
MT	Mersenne Twister	MSE	Mean Squared Error
NIST	National Institute of Standards and Technology	NL	Nonlinearity
NPCR	Number of Pixels Change Rate	PC	Personal Computer
PRNG	Pseudo-Random Number Generator	PSNR	Peak Signal-to-Noise Ratio
RAM	Random Access Memory	RGB	Red, Green, Blue
SAC	Strict Avalanche Criterion	SHA-2	Secure Hash Algorithm 2 (family)
SHA-256	Secure Hash Algorithm 256-bit	SHA-512	Secure Hash Algorithm 512-bit
SNR	Signal-to-Noise Ratio (in PSNR)	TEE	Trusted Execution Environment
TLS	Transport Layer Security	TPM	Tree Parity Machine
UACI	Unified Average Changing Intensity	UAV	Unmanned Aerial Vehicle
USC-SIPI	University of Southern California — Signal and Image Processing Institute	VM	Virtual Machine
XOR	Exclusive OR	XNOR	Exclusive NOR

## Literature review

The rapid growth of cloud-enabled multimedia services has spurred extensive research into image-specific cryptography that balances security, efficiency, and scalability. This literature review surveys key strands underpinning the proposed scheme: neural synchronization for key agreement via TPMs, pseudo-randomness sources including Mersenne Twister and cellular automata (notably Rule 30), DNA-inspired encoding and arithmetic for confusion/diffusion, and adaptive S-box construction assessed by standard cryptographic criteria. This literature review attempts to synthesize advances in each strand and highlight their limitations in cloud contexts, motivating the proposed multi-layer, TPM-driven scheme that diversifies entropy and strengthens resistance to statistical, linear, and differential attacks while remaining practical for real-time deployment.

The work in^24^ proposes a color image encryption scheme that combines three layers: a sine chaotic map for key-driven bitwise diffusion, an S-box derived from a fractional-order 4D hyperchaotic Chen system to introduce strong nonlinear confusion, and a hybrid DNA coding stage that interleaves reversible DNA arithmetic and logic under pseudo-random control.

The authors of^25^ propose an RGB image encryption scheme comprising three stages: a Rule 30 cellular automaton that generates a pseudo-random key for initial diffusion, a robust substitution box to enforce nonlinear confusion, and a Lorenz system-based key derived from a chaotic trajectory for a final diffusion layer. By combining CA-driven key-streams, an S-box built via transformation, modular inverses, and permutation, and a deterministic chaotic PRNG from the Lorenz system, the scheme targets strong confusion-diffusion properties while remaining lightweight and suitable for practical implementation.

In^26^, the authors propose a two-layer image encryption and transmission framework tailored for UAV-assisted relay networks in contested environments. The first layer applies a Mersenne Twister-seeded Genetic Algorithm that splits bytes into crossover and mutation paths, using an MT-derived S-box and XOR masking, while the second layer employs DNA coding with reversible arithmetic under MT-driven keys. The encrypted bit-stream is then channel coded (convolutional or LDPC), BPSK-modulated, and relayed via multi-hop UAV clusters using decode-and-forward, with equal-gain combining at the destination.

The authors of^27^ propose an image encryption scheme that integrates several discrete chaotic maps to achieve strong confusion and diffusion with minimal rounds. Their pipeline partitions RGB images into blocks, performs intra-block pixel shuffling via the 2D Hénon map, permutes blocks (rand-block operation), applies pixel-level distortion using the 1D Circle map, and finalizes diffusion with a bitwise XOR driven by the 2D Duffing map; decryption inverts these steps accordingly.

Further, the authors of^28^ propose a color image cipher that integrates hyperchaotic scrambling, SHA-512-driven key derivation, dynamic DNA operations, and Zaslavskii-map-based conditional shifts. The pipeline: (1) derive initial conditions from the plain image’s SHA-512; (2) scramble pixels using a 2D hyperchaotic 2D-LICM map; (3) generate a cover image via a combined logistic-tent map and build a mask by randomly sampling bits from the cover image’s SHA-512; (4) perform dynamic, per-pixel DNA encoding, DNA-XOR, and decoding with rules selected by chaotic sequences; (5) apply row/column circular shifts controlled by Zaslavskii-map outputs; and (6) execute repeated XOR diffusion rounds whose count and seeds are SHA-dependent.

While the authors of^29^ propose an image cipher that couples a novel algebraic confusion layer with chaos-driven diffusion. First, pixels are substituted via a substitution table constructed from an inverse left almost (LA) semigroup, introducing nonlinearity and breaking plaintext structure. Next, three logistic sequences are seeded from the Lorenz continuous chaotic system and applied per color layer (x*→ *R, y*→ *G, z*→ *B) through bitwise modulo-2 addition to diffuse changes across the image. Decryption inverts these steps.

The authors of^30^ propose a hybrid image cipher that couples a new two-dimensional Exponential Sine Map (2D-ESM) with cellular automata to achieve layered confusion and diffusion. The 2D-ESM generates pseudo-random sequences highly sensitive to initial conditions (validated via bifurcation diagrams, Lyapunov exponents, and NIST SP 800-22 tests) which are mapped into per-pixel confusion and diffusion matrices. Encryption proceeds by mod-257 value transformation driven by the diffusion matrix, additive confusion via the confusion matrix, matrix transposition, and a CA-derived masking stage (e.g., Rule-30-based evolution), with an accompanying SHA-256 hash used for integrity verification. Decryption inverts CA masking, permutation, and modular operations using the inverse diffusion

Moreover, the authors of^31^ propose a cellular-automata-driven image cipher that employs a hybrid one-dimensional CA configured with Rules 90 and 150 under null boundary conditions. Using a 24-cell array aligned with the 24 bits of an RGB pixel, the scheme treats the CA’s initial state (seed) as the symmetric key and encrypts/decrypts by XORing pixel components with the evolving CA state while advancing the CA via a unified update equation that merges the two rules. A rule pattern is selected to maximize cycle length (up to $2^n-1$), yielding pseudo-random key-streams for per-pixel processing.

In^32^, the authors propose an image protection scheme that combines TPM-based neural key agreement with a modified Vigenère cipher. Two parties independently initialize TPMs and synchronize their weights over a public channel by exchanging only common random inputs and output bits; when synchronization occurs, the identical weight vectors serve as a shared secret key without ever transmitting the key itself. For payload encryption, a Vigenère cipher is adapted to images by operating modulo 256 and mitigating periodic-key artifacts: the key value is multiplied by the row index (y-axis) so each image row uses a distinct effective shift. Experiments show low Pearson correlation between plaintext and ciphertext (near zero for the modified cipher) and that TPM synchronization time depends on network size (*K*, *N*, *L*), similarity of initial weights, and the randomness of input vectors (attractive vs. repulsive steps).

The work in^33^ introduces SLCA-Cipher, a lightweight image-encryption method aimed at IoT and other resource-limited devices. It builds encryption around 1D Cellular Automata Rule 90, where each cell updates via an XOR of its left and right neighbors, producing key-driven, pseudo-random evolution. The scheme converts a grayscale image to a binary grid, iteratively applies the CA updates to create strong scrambling and diffusion, and then converts the result back into an encrypted image.

In summary, Table 2 shows that prior image encryption algorithms have individually leveraged chaos/CA-driven key-streams, DNA-based confusion, adaptive S-boxes, or TPM synchronization, but rarely combine them in a cohesive, end-to-end design. In particular: (i) schemes with strong confusion-diffusion via CA/chaos and DNA typically rely on static or plaintext-derived seeds, leaving key establishment outside the cipher boundary; (ii) TPM-based works demonstrate certificate-free key agreement yet pair it with lightweight substitution (e.g., Vigenère), not with modern nonlinearity-gated S-boxes and multilayer diffusion; and (iii) multi-source PRNG diversification with independently derived seeds is largely absent, which leaves systems exposed to PRNG-specific biases and distinguishing attacks. Moreover, few studies validate seed-dependent S-boxes against composite criteria (NL, SAC, BIC, LAP, DAP) while integrating them with DNA and dual PRNGs under a neural key-exchange front end suitable for cloud settings. This gap motivates the proposed algorithm: a TPM-synchronized, session-oriented key establishment that deterministically seeds two orthogonal PRNGs (MT and Rule 30 CA) and drives quality-gated, seed-dependent S-boxes interleaved with reversible DNA operations. Thereby unifying secure key agreement with diversified entropy sources and multi-stage confusion-diffusion tailored for cloud-scale, real-time image protection.

**Table 2:** Comparison of image encryption schemes by core components, randomness sources, confusion, and diffusion mechanisms.

References	Core building blocks	Key/PRNG source	Confusion mechanism	Diffusion mechanism
^24^	Sine chaotic map; 4D hyperchaotic Chen (fractional-order) S-box; Hybrid DNA coding	Chaotic maps (sine; hyperchaos)	Nonlinear S-box (Chen-based); DNA ops (dynamic rules)	Bitwise masking via chaotic key-stream; DNA arithmetic/logic
^25^	Rule-30 CA key-stream; Algebraic S-box; Lorenz-system key	CA (Rule 30); Lorenz chaotic trajectory	Robust S-box (transform + inverses + permutation)	XOR/diffusion via CA and Lorenz PRNGs
^26^	MT-seeded Genetic Algorithm + S-box; DNA layer; channel coding	Mersenne Twister	GA crossover path + MT S-box	XOR masking; DNA addition; coded transmission
^27^	Hénon (2D), Circle (1D), Duffing (2D) maps	Chaotic maps	Intra-block shuffling; Block permutation	Pixel-level distortion; final XOR via Duffing
^28^	Hyperchaotic 2D-LICM scramble; Combined logistic-tent; Zaslavskii shifts; Dynamic DNA; SHA-512	SHA-512 from plaintext; Logistic-tent chaos	Dynamic DNA (per-pixel rules); Row/column shifts (Zaslavskii)	Repeated XOR rounds (SHA-dependent)
^29^	Inverse LA-semigroup S-box; Lorenz-seeded logistic streams	Lorenz *→ * per-channel logistic sequences	Algebraic substitution (LA-semigroup table)	Bitwise mod-2 addition per RGB (x*→ *R, y*→ *G, z*→ *B)
^30^	2D Exponential Sine Map (2D-ESM); CA mask; Matrix transpose	2D-ESM chaos (bifurcation, Lyapunov, NIST-validated)	Per-pixel confusion matrix; CA mask	Mod-257 diffusion matrix; inverse ops on decrypt
^31^	1D CA hybrid (Rules 90/150, null boundary)	CA seed (24-cell, long cycle)	XOR with evolving CA state (per-pixel, 24-bit alignment)	Key-stream evolution by CA update
^32^	Tree Parity Machine key agreement; Modified Vigenère (mod-256)	TPM-synchronized weights	Vigenère with row-scaled key (break periodicity)	Row-wise shift (mod-256) based diffusion
^33^	1D Cellular Automata (Rule 90); binary image grid; iterative round-based transform	Secret seed/key *→ * CA initialization; CA evolution acts as key-stream/key-grid generator	Key-dependent scrambling via iterative CA evolution (noise-like ciphertext appearance)	Iterative XOR-based neighbor update (Rule 90) yielding strong bit/pixel diffusion across rounds

## Preliminary ideas

### Tree parity machines

Tree Parity Machines are a type of neural network architecture used in machine learning, particularly in the field of cryptography for key exchange over a public channel^13^. They were introduced by Apostolico and Giancarlo in 1990. A Tree Parity Machine is a type of feedforward neural network with three layers: an input layer, a hidden layer, and an output layer. The input and output layers consist of binary values, while the hidden layer consists of neurons with continuous weights. The unique property of TPMs is that two or more TPMs can be synchronized over a public channel. This is done by repeatedly sending random input vectors to the TPMs and updating their weights according to a learning rule. Over time, the weights of the TPMs converge, effectively synchronizing them. This property makes TPMs useful for secure key exchange: once the TPMs are synchronized, their weights can be used as a shared secret key. Tree Parity Machines are robust to various types of attacks, including geometric and flipping attacks. They have been used in several cryptographic systems and have also been used to model biological processes, such as the synchronization of fireflies^34^. In the implemented TPM, the adopted parameter values are

$$ N=100,\qquad K=10,\qquad L=10, $$

where, according to Algorithm 1, *N* denotes the number of hidden neurons, *K* denotes the number of input neurons connected to each hidden neuron, and *L* denotes the range of each weight, i.e.,

$$ W_{i,j}\in \{-L,\ldots ,0,\ldots ,+L\}. $$

These values are selected to provide a practical balance between security strength and synchronization efficiency. Since the TPM contains *N× K* trainable weights, the above choice yields

$$ N\times K=100\times 10=1000 $$

weights in the synchronized weight matrix. In addition, each weight can take *2L+1=21* possible values. Therefore, the weight-space size of the TPM alone is

$$ (2L+1)^{N\times K}=21^{1000}\approx 2^{4392}, $$

which indicates a very large search space and thus strengthens resistance against brute-force recovery and passive attacker synchronization attempts.

The steps of the synchronization process are outlined as follows:

1. The size of the TPM is chosen by the two parameters *K* and *N* (input layer and hidden layer, respectively).
2. Random input vectors of size *n* are chosen and sent to both TPMs.
3. Each TPM computes an output value based on its current weights and the input vector using Algorithm 1.
4. The TPMs exchange their output values. If the outputs match, both TPMs modify their weights according to the input vector by the Hebbian rule, as in Algorithm 2. If the outputs do not match, no changes are made.
5. This process is repeated until the weights of the TPMs match, meaning they are synchronized.

The influence of the three parameters can be summarized as follows. Increasing *N* increases the number of hidden neurons, while increasing *K* increases the number of inputs associated with each hidden neuron. In both cases, the total number of weights *N× K* grows, which enlarges the TPM state space and increases the difficulty of inferring the synchronized weight vector from the exchanged public information. Increasing *L* enlarges the number of admissible values of each weight and therefore further expands the search space. However, larger values of *N*, *K*, and *L* also tend to increase the synchronization time, because the two communicating TPMs must converge in a higher-dimensional weight space and over a wider weight interval. For this reason, (*K*, *N*, *L*) are treated in this work as security parameters that directly affect both the resistance to known TPM attacks and the convergence behavior of the legitimate parties.

To quantify this trade-off, synchronization experiments were conducted using the Hebbian update rule with the above parameter setting. Over 20 independent synchronization trials, the number of iterations required to reach identical weights ranged from 508 to 1139, with a mean of 763, a median of 738, and a standard deviation of *± 132*. Since each synchronization iteration corresponds only to the exchange of the TPM output *τ *, this overhead remains small and is incurred only once during session establishment. Hence, the adopted setting *(N,K,L)=(100,10,10)* is selected as a suitable compromise: it provides a very large TPM weight space while maintaining an acceptable synchronization cost for the intended cloud image-encryption scenario.

**Algorithm 1:**
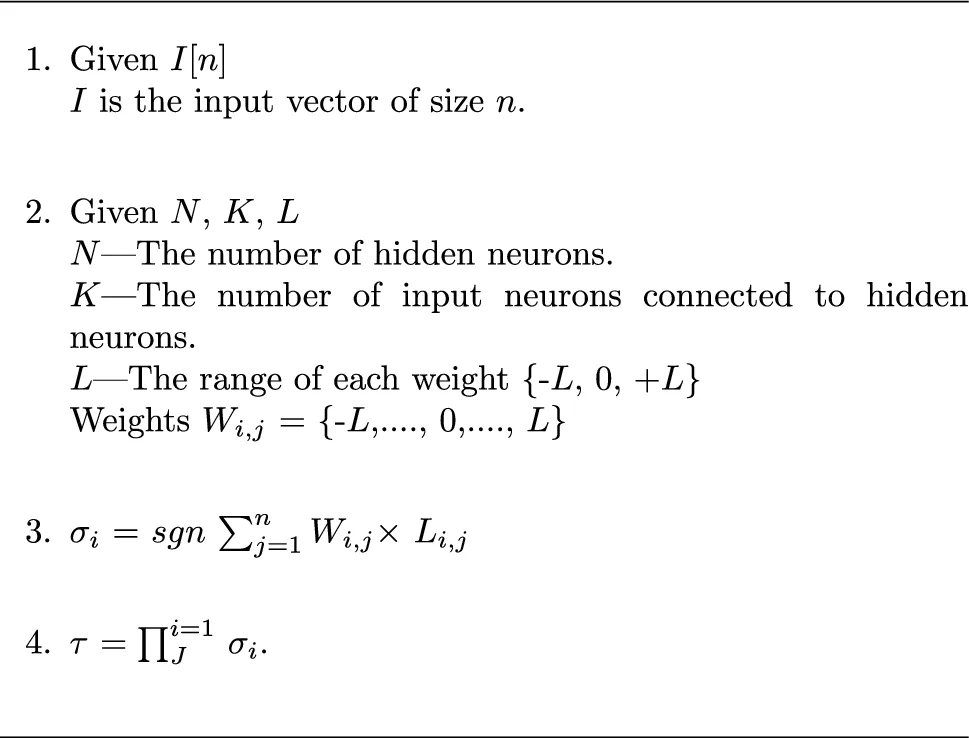
Tree Parity Machine, as adopted from^35^.

To further evaluate the practicality of the TPM stage for real-time cloud applications, the synchronization cost is measured for the same TPM configuration adopted in this work, namely *(N,K,L)=(100,10,10)*, using the Hebbian update rule. Over 20 independent synchronization trials, the number of iterations required to reach identical weights ranged from 508 to 1139, with a mean of 763, a median of 738, and a standard deviation of *± 132*. These results indicate that the synchronization overhead remains limited in practice, since each iteration requires only the exchange of the TPM output *τ * over the public channel. Moreover, synchronization is performed only once during session establishment, after which the synchronized weight vectors are used to derive the keys for the subsequent encryption stages. Therefore, the TPM synchronization cost is considered acceptable for the intended real-time cloud image-transmission scenario.

**Algorithm 2:**
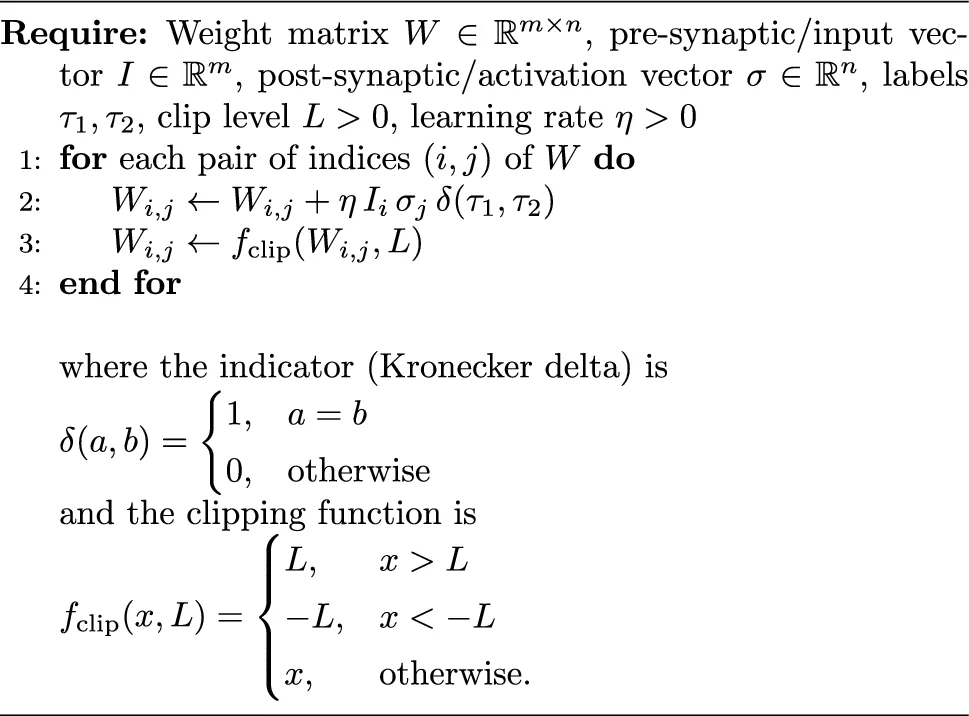
Hebbian rule adapted from^35^

###  Mersenne twister

The Mersenne Twister (MT) is a widely used pseudo-random number generator introduced by Makoto Matsumoto and Takuji Nishimura in 1997. It produces sequences of 32-bit integers using a linear recurrence (often described in terms of LFSR-like operations) together with a tempering function that improves the distribution of the generated values^36^. In an LFSR, the register contents are shifted and selected bits are combined using exclusive-OR (XOR) operations to generate subsequent bits; similarly, the MT updates an internal state of 624 32-bit words and then applies tempering before output.

The MT is based on a large Mersenne prime of the form $2^p-1$, where *p* is also prime. The commonly used MT19937 variant is designed to have a very long period of $2^{19937}-1$, i.e., the maximum number of values that can be generated before the sequence repeats^37^. While this long period and the tempering step provide strong statistical behavior, it is important to note that MT is *not* cryptographically secure by design: due to its linear structure, if a sufficiently long segment of MT output is exposed, an attacker can potentially reconstruct the internal state and predict future outputs. Therefore, MT should not be used as a stand-alone key-stream generator in adversarial settings.

In the proposed scheme, the MT is used only as a supporting randomness source within a layered construction rather than as an independent security primitive. Specifically, the MT is deterministically seeded from secret material (derived from the synchronization process) and is not seeded with attacker-controlled or low-entropy values; moreover, its output is not relied upon alone but is combined with other nonlinear and diffusion stages in the overall pipeline. This design choice reduces the practical risk associated with MT’s non-cryptographic nature, but it is explicitly acknowledged that statistical strength and long period do not imply cryptographic security. For high-assurance deployments, MT can be replaced with a standard CSPRNG (e.g., AES-CTR or ChaCha20) without changing the architecture of the proposed method^38^.

**Fig. 1:**
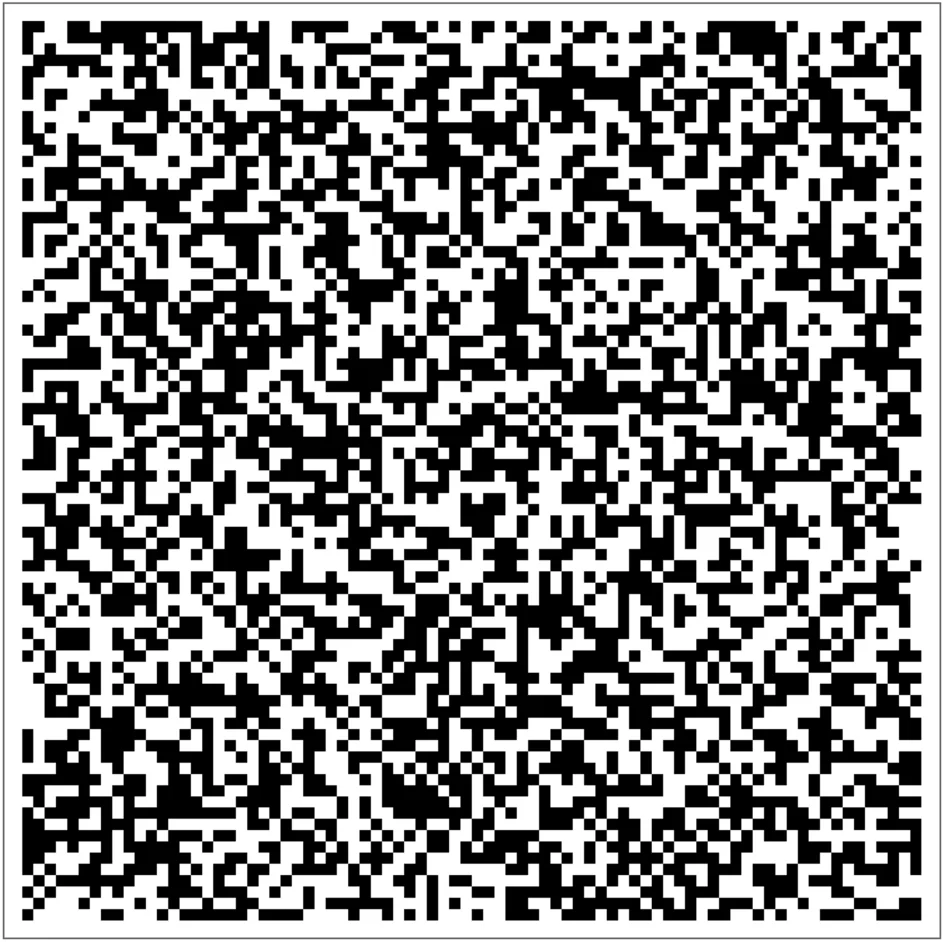
Array plot for Mersenne Twister with a seed 4567 of size *80× 80*.

In Fig. 1, a seed of value 4567 is given to the MT and the output is plotted as a 2D array of bits of size *80× 80*.

###  DNA coding

DNA sequences consist of four deoxyribonucleic acids: A (adenine), T (thymine), C (cytosine), and G (guanine). These nucleotides form complementary pairs, with A pairing with T and C pairing with G. When using DNA sequences for binary numbers, 0 and 1 are complementary. Image encryption using DNA coding is a technique that utilizes the complexity and randomness of DNA sequences to encrypt digital images. The process involves converting the image into a binary format and breaking it down into small blocks of data. Each block is assigned a corresponding DNA sequence using a DNA coding technique, which is generated randomly using a random number or pseudo-random number generator^24^. In such a case, each block is basically a base-4 number^39^.

To assign DNA sequences to binary values of an image, the 8-bit gray values are split into pairs of 00, 01, 10, 11, and assigned to the DNA base pairs according to a specific rule. There are 24 possible permutations, but only 8 are possible and are displayed in Table 3. The first rule assigns

1 $$\begin{aligned} {(00 \rightarrow A),(01 \rightarrow C),(10 \rightarrow G),(11 \rightarrow T)}. \end{aligned}$$

**Fig. 2:**
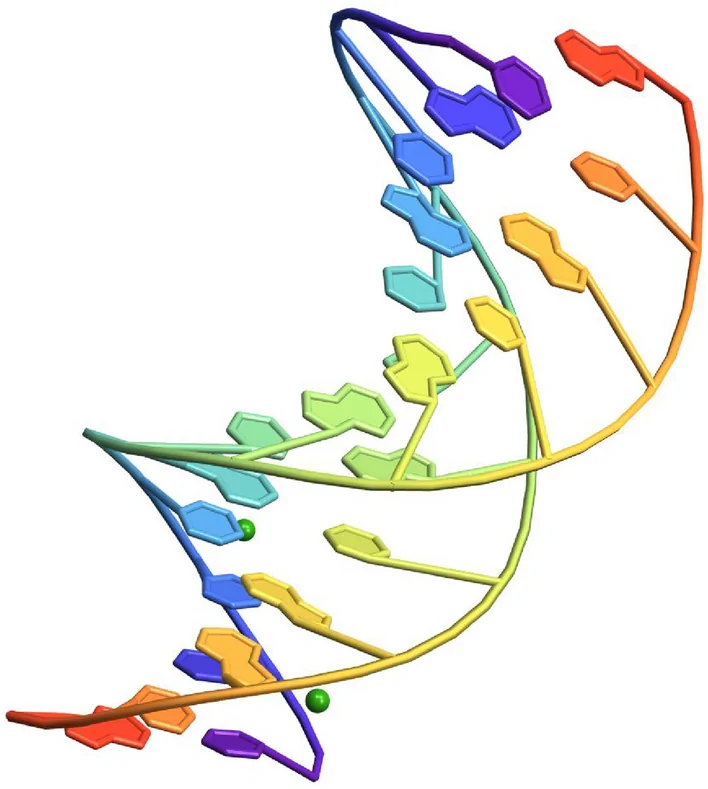
An example DNA strand.

In addition to DNA rules, reversible DNA operations, such as addition and subtraction, which reverse each other, and XOR or XNOR, are used for encryption. These operations are carried out in accordance with a specific table, as shown in Table 4. A visual representation of a DNA strand plot is shown in Fig. 2.

The reversibility of the proposed DNA-based transformation follows from two facts. First, each DNA encoding rule in Table 3 defines a bijection between the four binary pairs $\{00,01,10,11\}$ and the four nucleotides $\{A,T,C,G\}$, which guarantees unique encoding and decoding. Second, the DNA arithmetic table in Table 4 is constructed so that subtraction is the inverse operation of addition for every ordered nucleotide pair. Therefore, for any DNA symbols $X,Y \in \{A,T,C,G\}$, the operation

2 $$\begin{aligned} Z = \textrm{Add}_{\textrm{DNA}}(X,Y) \end{aligned}$$

admits the unique inverse

3 $$\begin{aligned} X = \textrm{Sub}_{\textrm{DNA}}(Z,Y). \end{aligned}$$

For example, from Table 4, *A + G = G*. Applying the inverse operation gives *G - G = A*, which recovers the original symbol exactly. Similarly, *T + C = G* and *G - C = T*. This confirms the perfect reversibility of the adopted DNA arithmetic. In the proposed algorithm, dynamic DNA coding is carried out, in such a manner that depending on the image dimensions, a DNA coding rule is applied, as shown in Table 5.

Thus, the DNA addition/subtraction stage is lossless and fully reversible, ensuring exact recovery of the original encoded sequence during decryption. In the same manner, the DNA XOR and DNA XNOR operations are reversible under the adopted rule table because each ordered input pair produces a unique output symbol. Therefore, once the operand and the encoding rule are known, the original symbol can be uniquely retrieved during decryption by applying the corresponding inverse table lookup. Hence, the DNA addition, subtraction, XOR, and XNOR stages used in the proposed method are all lossless.

**Table 3:** Permutations of DNA to bits assignment.

Rule	1	2	3	4	5	6	7	8
*A*	00	00	11	10	01	10	01	11
*T*	11	11	00	01	10	01	10	00
*G*	10	01	10	11	00	00	11	01
*C*	01	10	01	00	11	11	00	10

**Table 4:** Arithmetic and logic operations to be performed on DNA {Addition, Subtraction, XOR, XNOR}.

	*A*	*T*	*C*	*G*
*A*	{*A*,*A*,*A*,*G*}	{*T*,*G*,*T*,*C*}	{*C*,*C*,*C*,*T*}	{*G*,*T*,*G*,*A*}
*T*	{*T*,*T*,*T*,*C*}	{*C*,*A*,*A*,*G*}	{*G*,*G*,*G*,*A*}	{*A*,*C*,*C*,*T*}
*C*	{*C*,*C*,*C*,*T*}	{*G*,*T*,*G*,*A*}	{*A*,*A*,*A*,*G*}	{*T*,*G*,*T*,*C*}
*G*	{*G*,*G*,*G*,*A*}	{*A*,*C*,*C*,*T*}	{*T*,*T*,*T*,*C*}	{*C*,*A*,*A*,*G*}

**Table 5:** DNA coding rule to apply depending on image dimensions.

Image dimensions	Rule to apply
*16 × 16*	1
*32 × 32*	2
*64 × 64*	3
*128 × 128*	4
*256 × 256*	5
*512 × 512*	6
*1024 × 1024*	7
*2048 × 2048*	8

###  Cellular automata

Cellular automata are structures made up of cells arranged in a grid that follow simple rules to produce patterns. Each cell in a two-dimensional grid has a binary state of either 0 or 1, which is determined by its neighboring cells based on a set of rules^25^. The Rule 30 cellular automaton is used in the encryption process, providing a formula for determining the next state of each cell. The most significant property of cellular automata is their ability to generate complex patterns using simple mathematical rules. These patterns are useful for scrambling and encrypting image data, rendering it unreadable without the proper decryption key^40^. The formula for determining the subsequent state of a cell is as follows:

4 $$\begin{aligned} s_i(t+1) = s_{i-1}(t) \oplus (s_i(t)+ s_{i+1}(t)), \end{aligned}$$

**Fig. 3:**
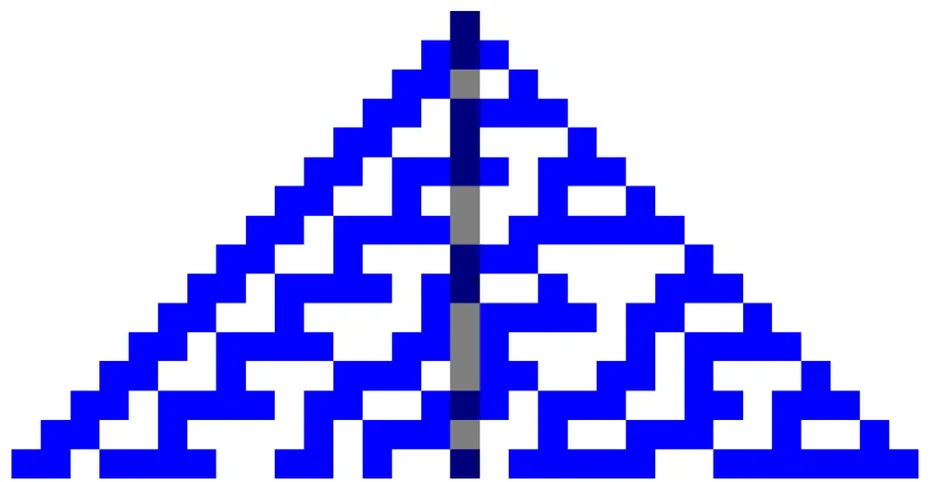
The initial 15 steps of the Rule 30 Cellular automaton.

$s_i(t)$ represents the current state of the cell *i* at time *t*, and $s_i(t+1)$ represents its next state at time *t+1*. The *⊕ * symbol represents the exclusive OR (XOR) operation, which returns a 1 if the two inputs are different, and a 0 if they are the same. The first 15 iterations of the Rule 30 CA are demonstrated in Fig. 3 can be considered as a PRNG, as the center column satisfies the characteristics of a randomly generated bit-stream^41^. Specifically, the center column of Fig. 3 reveals that the resulting bit-stream is (1, 1, 0, 1, 1, 1, 0, 0, 1, 1, 0, 0, 1, 0, 1). Rule 30 is adopted in this work because it provides a practical balance between implementation simplicity, computational efficiency, and strong pseudo-random behavior. In comparison with many other elementary cellular automata rules that generate regular, symmetric, or more predictable patterns, Rule 30 is well known for producing highly irregular and chaotic-looking sequences even from simple initial conditions. This property makes it particularly suitable for image encryption, where lightweight generation of complex bit-streams is required to enhance confusion and diffusion^42^. Nevertheless, Rule 30 is deterministic and is not claimed here as a standalone cryptographically secure pseudo-random generator. Its randomness quality may vary depending on the selected output cell and extraction strategy, and therefore the security of the proposed scheme does not rely solely on Rule 30. Instead, Rule 30 is employed as an auxiliary randomness-enhancing component within the overall hybrid encryption framework.

## Methodology of the proposed TPM image encryption scheme

### Encryption scheme

In this section, the proposed image encryption scheme is implemented in a number of steps, as follows:

1. First Encryption Layer:
  - An image of appropriate dimensions *M× N* is chosen and its pixels are converted into a 1D bit-stream, $I_B$.
  - An initial weight vector is chosen for synchronization between the sender and the receiver to get *finalWeightVector*1.
  - The *finalWeightVector*1 is given as an input to the hash function (SHA-512) to generate the seed $S_1$:
    5 $$\begin{aligned} S_1 = SHA-512(final Weight Vector 1). \end{aligned}$$
  - $S_1$ is given as a seed to MT to generate an encryption key $K_1$.
    6 $$\begin{aligned} MT(S_1) = K_1, \end{aligned}$$
  - $I_B$ is XOR-ed with $K_1$ to generate the bit-stream $B_1$:
    7 $$\begin{aligned} B_1 = I_B \oplus K_1. \end{aligned}$$
  - Every eight bits of $K_1$ are grouped together, forming an array of integers $A_1$ which ranges from 0 to 255.
  - $A_1$ is then used to generate $S-box_1$ using the MT and Algorithm 3, with the desired threshold:
    8 $$\begin{aligned} S-box_1 = Algorithm~3 (A_1, 10, MT), \end{aligned}$$
  - $S-box_1$ is applied to $B_1$ to generate the encrypted image $I_2$ with bit-stream $B_2$:
    9 $$\begin{aligned} I_2 = S-box_1 (B_1), \end{aligned}$$
    10 $$\begin{aligned} B_2 = bitStream (I_2). \end{aligned}$$
2. Second Encryption Layer:
  - Key $K_1$ is given as a seed to the MT to create the second key $K_2$, then it is converted to DNA to get $K_{2DNA}$:
    11 $$\begin{aligned} K_2 = MT(K_1), \end{aligned}$$
    12 $$\begin{aligned} DNA(K_2) = K_{2DNA}. \end{aligned}$$
  - The image bits $B_2$ are also converted to DNA to generate $B_{2DNA}$.
    13 $$\begin{aligned} DNA(B_2) = B_{2DNA}. \end{aligned}$$
  - DNA addition is preformed between $K_{2DNA}$ and $B_{2DNA}$ giving DNA of $B_{3DNA}$ which formulates an encrypted image $I_3$:
    14 $$\begin{aligned} B_{3DNA} = Add_{DNA}(K_{2DNA}, B_{2DNA}). \end{aligned}$$
3. Third Encryption Layer:
  - The *finalWeightVector*2 is given as an input to the hash function (SHA-512) to generate the seed $S_3$:
    15 $$\begin{aligned} S_3 = SHA-512(final Weight Vector 2). \end{aligned}$$
  - $S_3$ is given as a seed to Rule30CA to get an encryption key $K_3$.
    16 $$\begin{aligned} Rule30CA(S_3) = K_3. \end{aligned}$$
  - Every eight bits of $K_3$ are grouped together to form an array of integers $A_2$ which ranges from 0 to 255.
  - $A_2$ is used to generate $S-box_2$ using Rule30CA and Algorithm 3, with the desired threshold:
    17 $$\begin{aligned} S-box_2 = Algorithm~3 (A_2, 10, Rule30CA). \end{aligned}$$
  - $B_{3DNA}$ is then mapped into bits as $B_3$ and then XOR-ed with $K_3$ to generate the bit-stream $B_4$:
    18 $$\begin{aligned} B_4 = B_3 \oplus K_3. \end{aligned}$$
  - $S-box_2$ is applied on $B_4$ to generate the final encrypted image *I'*:
    19 $$\begin{aligned} I' = S-box_2 (B_4). \end{aligned}$$

**Algorithm 3:**
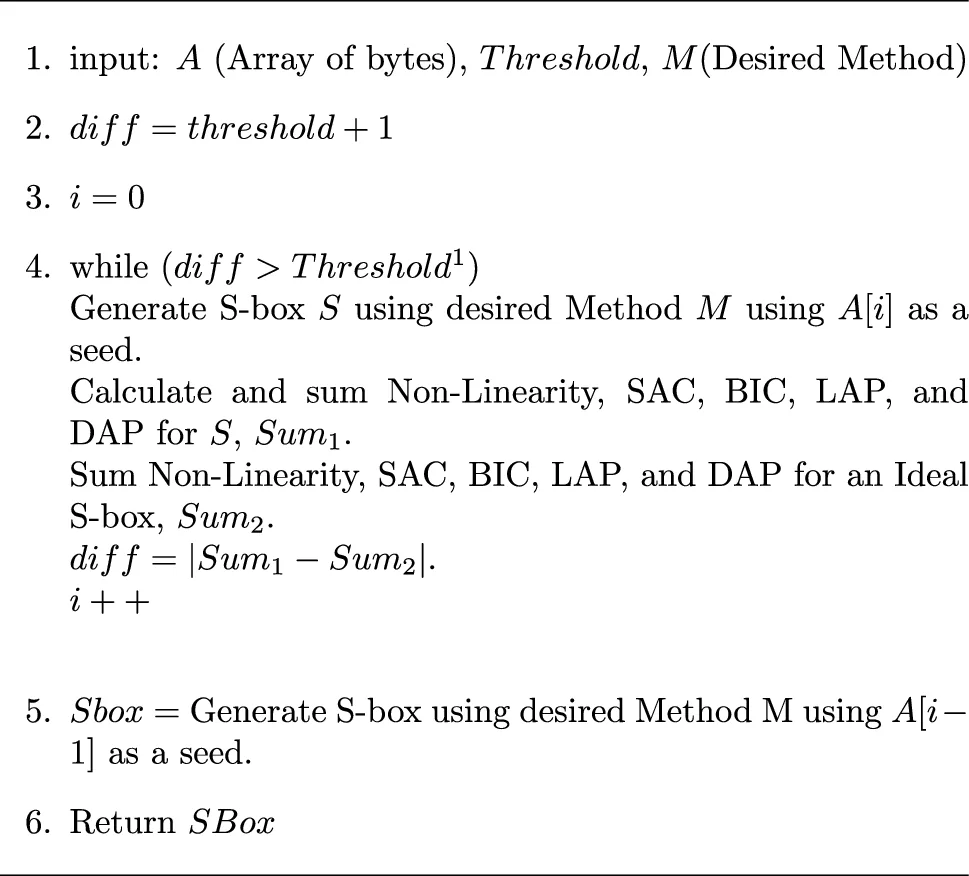
S-box Generation.

**Table 6:** S-box 1 generated from MT $SBox_{\text {MT}}$.

175	219	68	3	232	108	60	194	137	15	65	227	89	54	24	94
41	136	224	201	166	132	7	211	36	117	247	233	56	123	162	12
85	20	21	157	9	167	109	160	241	192	154	30	218	70	87	240
29	62	138	28	5	120	172	37	39	129	100	174	58	72	238	113
17	234	13	198	77	25	159	49	119	84	4	223	242	163	32	131
53	230	42	114	35	134	112	144	196	156	177	91	22	253	140	97
81	44	231	179	88	50	124	203	204	1	202	186	111	71	46	107
141	130	190	18	95	139	26	76	34	121	127	47	248	74	14	161
146	246	252	10	149	237	197	105	51	173	106	222	43	78	191	185
243	187	104	80	59	145	249	180	255	115	152	45	215	217	86	64
79	158	236	122	184	171	206	181	245	176	239	93	98	165	38	188
209	66	250	125	69	235	31	82	75	254	63	169	189	251	213	220
153	128	168	40	6	11	244	150	193	164	212	16	27	0	116	90
195	214	103	148	33	83	170	143	48	207	52	199	55	226	96	67
110	182	61	101	200	99	208	135	225	151	57	183	126	2	228	205
118	23	19	178	147	155	73	142	210	133	221	216	229	102	92	8

**Table 7:** S-box 2 generated from Rule30 CA $SBox_{\text {CA}}$.

106	32	110	224	16	170	186	182	104	51	72	70	231	62	117	109
181	197	99	142	98	108	158	221	95	53	234	214	120	8	77	203
247	50	128	137	229	103	83	187	161	87	216	124	86	39	28	15
246	31	176	74	168	160	7	166	65	49	204	165	79	23	174	1
171	102	177	230	57	156	183	235	239	112	3	218	20	91	90	220
127	155	26	202	179	189	175	129	147	2	140	47	200	232	242	251
212	97	159	237	114	35	225	205	253	73	223	178	27	122	52	34
81	58	61	25	153	240	254	17	0	149	60	46	116	211	201	43
93	248	11	101	94	162	148	209	82	24	113	67	138	208	36	196
241	22	84	219	126	157	134	76	217	133	121	45	185	40	227	19
206	245	125	115	42	135	190	44	172	85	63	198	4	180	88	188
215	244	163	243	136	164	12	131	145	6	236	141	48	207	92	78
29	111	255	119	169	192	252	75	10	238	191	226	150	13	66	139
213	80	199	33	132	21	222	194	14	249	228	30	233	100	144	118
184	37	195	5	56	69	38	71	151	173	107	54	89	250	41	18
64	143	9	105	154	210	55	68	130	193	146	59	152	123	167	96

**Fig. 4:**
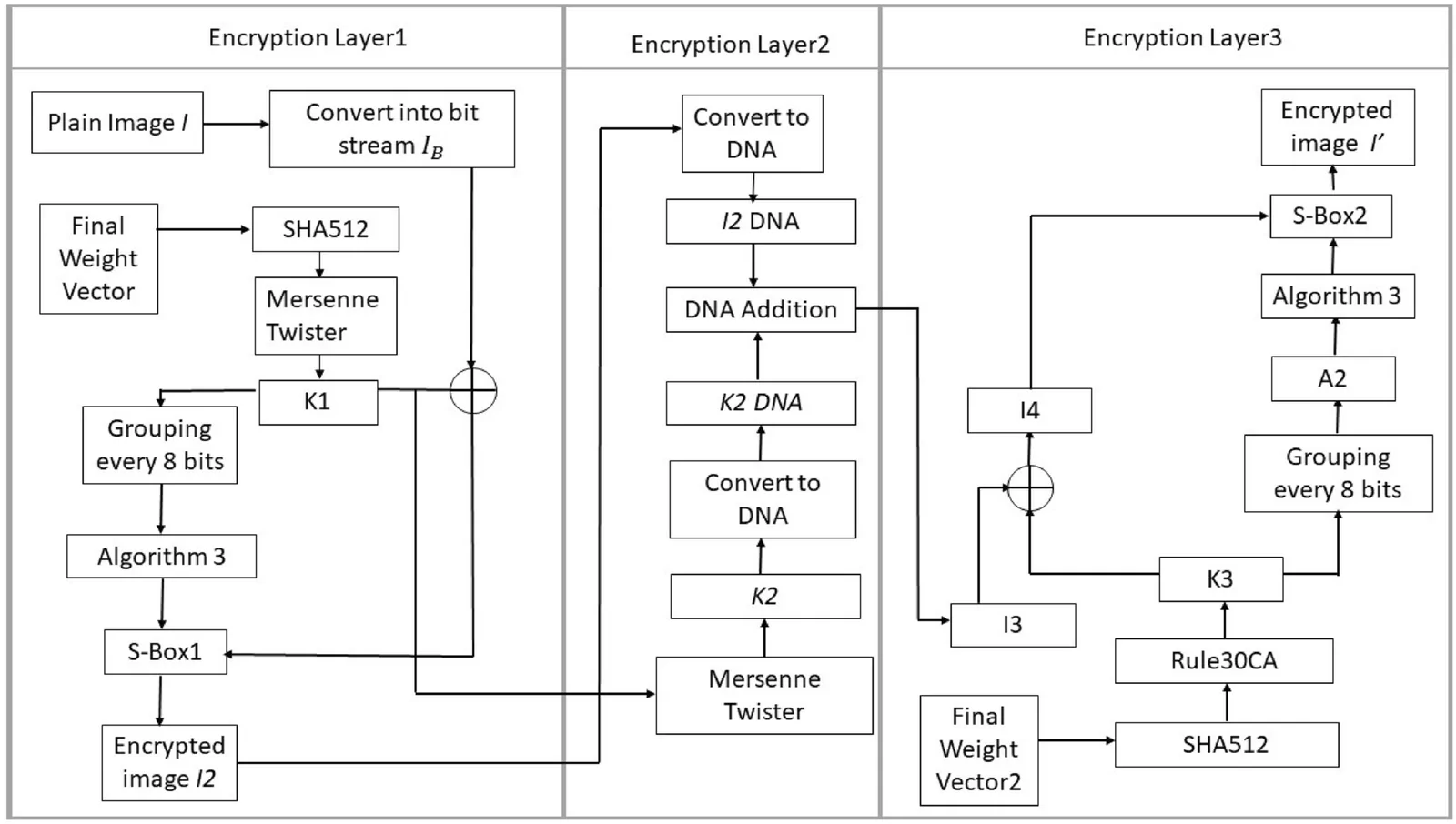
A flow chart demonstrating the 3-level encryption process.

A visual representation for the encryption scheme as a flow chart is provided in Fig. 4 (Tables 6 and 7).

### Decryption scheme

The decryption scheme is implemented in a reverse manner of the encryption scheme as follows:

1. First Decryption Layer:
  - The encrypted image *I'* is converted into a bit-stream $I'_B$:
    20 $$\begin{aligned} I'_B = bitStream(I'). \end{aligned}$$
  - FinalWeightVector2 is given to the hash function (SHA-512) to create the seed $S_3$:
    21 $$\begin{aligned} S_3 = SHA-512(Final Weight Vector 2). \end{aligned}$$
  - $S_3$ is given as seed to Rule30CA to generate $K_3$:
    22 $$\begin{aligned} Rule30CA(S_3) = K_3. \end{aligned}$$
  - Every eight bits of $K_3$ are grouped together to form an array of integers $A_2$ ranging from 0 to 255.
  - $A_2$ is then used to generate the S-box $S-box_2$ using Rule30CA and Algorithm 3, with the desired threshold:
    23 $$\begin{aligned} S-box_2 = Algorithm~3(A_2, 10, Rule30CA). \end{aligned}$$
  - $Reverse S-box_2$ is applied to $I'_B$ then XOR-ed with $K_3$ to create the first decrypted image $B_1$:
    24 $$\begin{aligned} B_1 = Reverse S-box_2(I'_B) \oplus K_3. \end{aligned}$$
2. Second Decryption Layer:
  - $K_1$ is a given as a seed to MT to create $K_2$ and then converted to DNA to get $K_{2DNA}$:
    25 $$\begin{aligned} K_2 = MT(K_1), \end{aligned}$$
    26 $$\begin{aligned} DNA(K_2) = K_{2DNA}. \end{aligned}$$
  - $B_1$ is converted into DNA. Then DNA subtraction is performed between $K_{2DNA}$ and $B_1$. The result is then restored into bits to generate the second decrypted image in bit-stream $B_2$.
    27 $$\begin{aligned} B_{2DNA} = Subtract_{DNA}(B_{1DNA}, K_{2DNA}), \end{aligned}$$
    28 $$\begin{aligned} B_2 = bitStream(B_{2DNA}), \end{aligned}$$
3. Third Decryption Layer:
  - FinalWeightVector1 is given to the hash function (SHA-512) to create the seed $S_1$:
    29 $$\begin{aligned} S_1 = SHA-512(Final Weight Vector 1). \end{aligned}$$
  - $A_1$ is then used to generate the S-box $S-box_1$ using MT and Algorithm 3 with the desired threshold:
    30 $$\begin{aligned} S-box_1 = Algorithm~3(A_2, 10, MT). \end{aligned}$$
  - Reverse $S-box_1$ is applied on the bit-stream $B_2$. Then the result is XOR-ed with $K_1$ to generate the final decrypted Image *I*:
    31 $$\begin{aligned} I = Reverse S-box_1(B_2) \oplus K_1. \end{aligned}$$

**Fig. 5:**
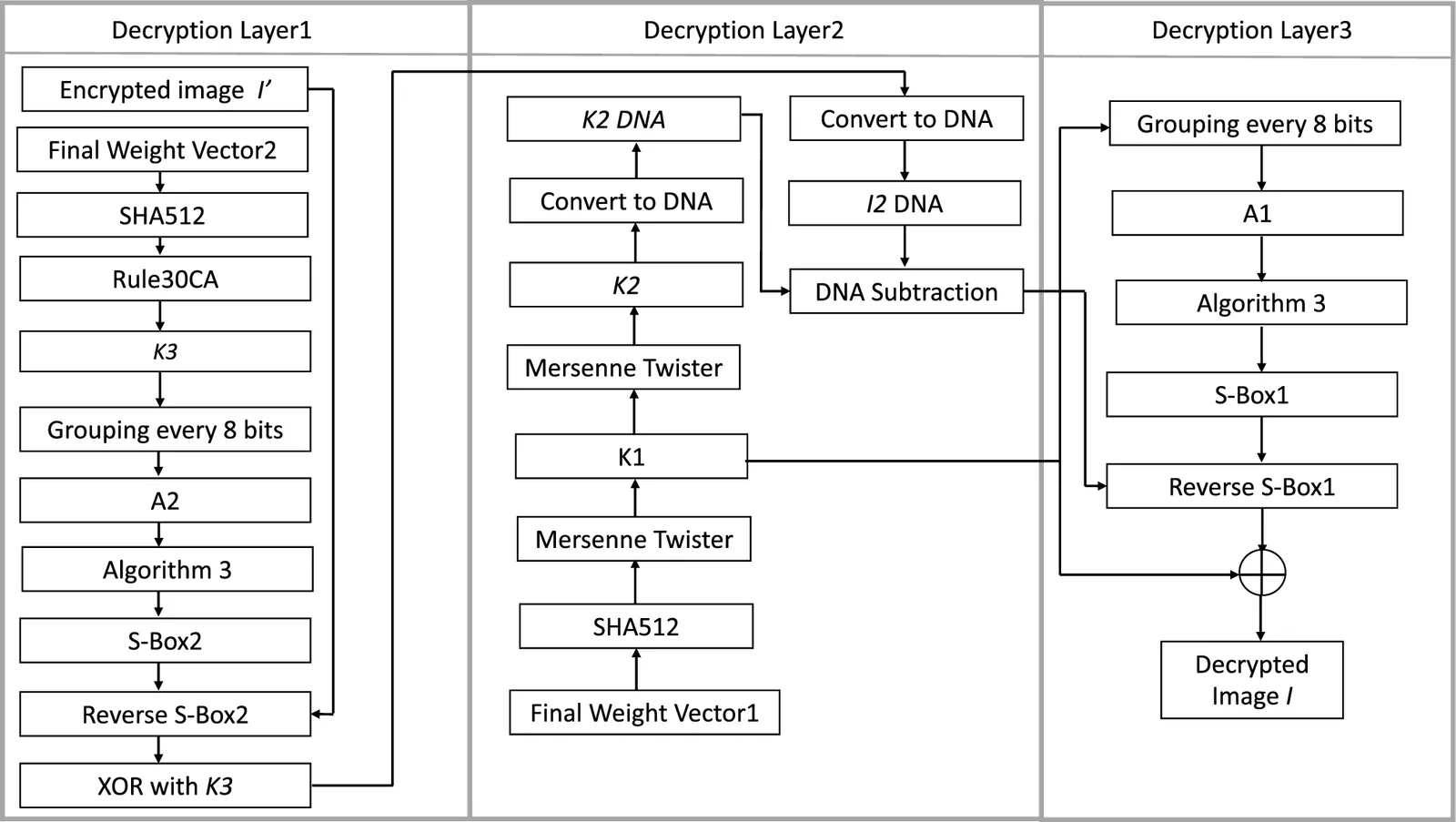
A flow chart demonstrating the 3-level decryption process.

A visual representation for the decryption scheme as a flow chart is provided in Fig. 5.

### Key confirmation/authentication

After both communicating parties complete TPM synchronization and derive the session key material, a key-confirmation step is performed by exchanging an authentication tag (e.g., HMAC-SHA-512) computed over a hash of the synchronization transcript. If verification fails, the session is aborted. This step prevents MITM attacks by ensuring both endpoints derived the same key and that the transcript is not modified.

## Performance analysis and evaluation

This section includes a full analysis of the proposed encryption scheme based on widely known metrics summarized in Table 8. The utilized PC has the following specifications: 2.7 GHz Intel^®^ Core^TM^ i7, 8 GB. All images in use are sourced from the USC-SIPI image database^43^, and resized to *256× 256* unless otherwise specified. The following metrics are computed:

- *Mean squared error (MSE):* A large MSE between the original and encrypted images is preferred, as it reflects substantial distortion introduced by the encryption algorithm.
- *Peak signal-to-noise ratio (PSNR):* A low PSNR value between the original and encrypted images is desirable, signifying minimal similarity and therefore stronger encryption resistance.
- *Mean absolute error (MAE):* Higher MAE values indicate significant differences in pixel intensities caused by encryption, which is a desirable characteristic of a secure encryption scheme.
- *Entropy:* Entropy quantifies the level of randomness in an image. *Global entropy* measures the overall distribution of pixel intensities, while *local entropy* evaluates complexity within small neighborhoods ^44^. For encrypted images, high global entropy values (close to 8 for 8-bit grayscale images) indicate strong randomness and uniform pixel distribution.
- *Discrete fourier transform (DFT):* After encryption, the frequency spectrum of the image should appear flat and noise-like, without any visible peaks or patterns, indicating the absence of exploitable frequency information.
- *Pixel correlation coefficient (PCC):* A secure encryption scheme produces encrypted images with correlation coefficients close to zero between adjacent pixels, demonstrating the removal of spatial dependencies.
- *Number of pixels change rate (NPCR):* High NPCR values (typically above 99%) indicate strong sensitivity to small changes in the plaintext image, making the algorithm resistant to differential attacks.
- *Unified average changing intensity (UACI):* For grayscale images, ideal UACI values are around 33.35%, reflecting substantial average intensity variations between two encrypted outputs generated from slightly different plaintexts.

**Table 8:** Mathematical formulations of the performance metrics employed for assessing image encryption.

Metric	Descriptive equation(s)
MSE	$MSE = \frac{\sum _{i=0}^{M-1} \sum _{j=0}^{N-1} (I_{i,j} - I'_{i,j})^2}{M \times N}.$
PSNR	$PSNR = 10 \log _{10} \left( \frac{I_{max}^2}{MSE}\right) , \quad I_{max} = 255.$
MAE	$MAE = \frac{1}{M \times N} \sum _{i=0}^{M-1} \sum _{j=0}^{N-1} |P_{i,j} - E_{i,j}|.$
Entropy	$H(m) = \sum _{i=1}^M p(m_i) \log _2 \frac{1}{p(m_i)}.$
DFT	$F(k,l) = \sum _{i=0}^{N-1} \sum _{j=0}^{N-1} f(i,j) e^{-i 2\pi \left( \frac{ki}{N} + \frac{lj}{N}\right) }.$
PCC	$\rho (x,y) = \frac{\text {cov}(x,y)}{\sqrt{\sigma (x)} \sqrt{\sigma (y)}},$ where $\text {cov}(x,y) = \frac{1}{N} \sum _{i=1}^N (x_i - \mu _x)(y_i - \mu _y), \quad \sigma (x) = \frac{1}{N}\sum _{i=1}^{N}(x_i-\mu (x))^2, \quad \mu (x)= \frac{1}{N}\sum _{i=1}^{N}(x_i).$
NPCR	$NPCR = \frac{\sum _{x=1}^M \sum _{y=1}^N D(x,y)}{M \times N} \times 100,$ where $$ D(x,y) = {\left\{ \begin{array}{ll} 0, & I(x,y) = I'(x,y), \\ 1, & \text {otherwise}. \end{array}\right. } $$
UACI	$UACI = \frac{1}{M \times N} \sum _{x=1}^M \sum _{y=1}^N \frac{|I_1(x,y) - I_2(x,y)|}{255} \times 100.$

### Visual and histogram analyses

Histogram analysis is a technique used in image processing and analysis to examine the distribution of pixel values in an image. This technique involves plotting the frequency of each pixel value in the image and analyzing the resulting histogram. In image encryption, histogram analysis is used as a metric to evaluate the effectiveness of the encryption algorithm by comparing the histogram of the original image with the histogram of the encrypted image.

**Fig. 6:**
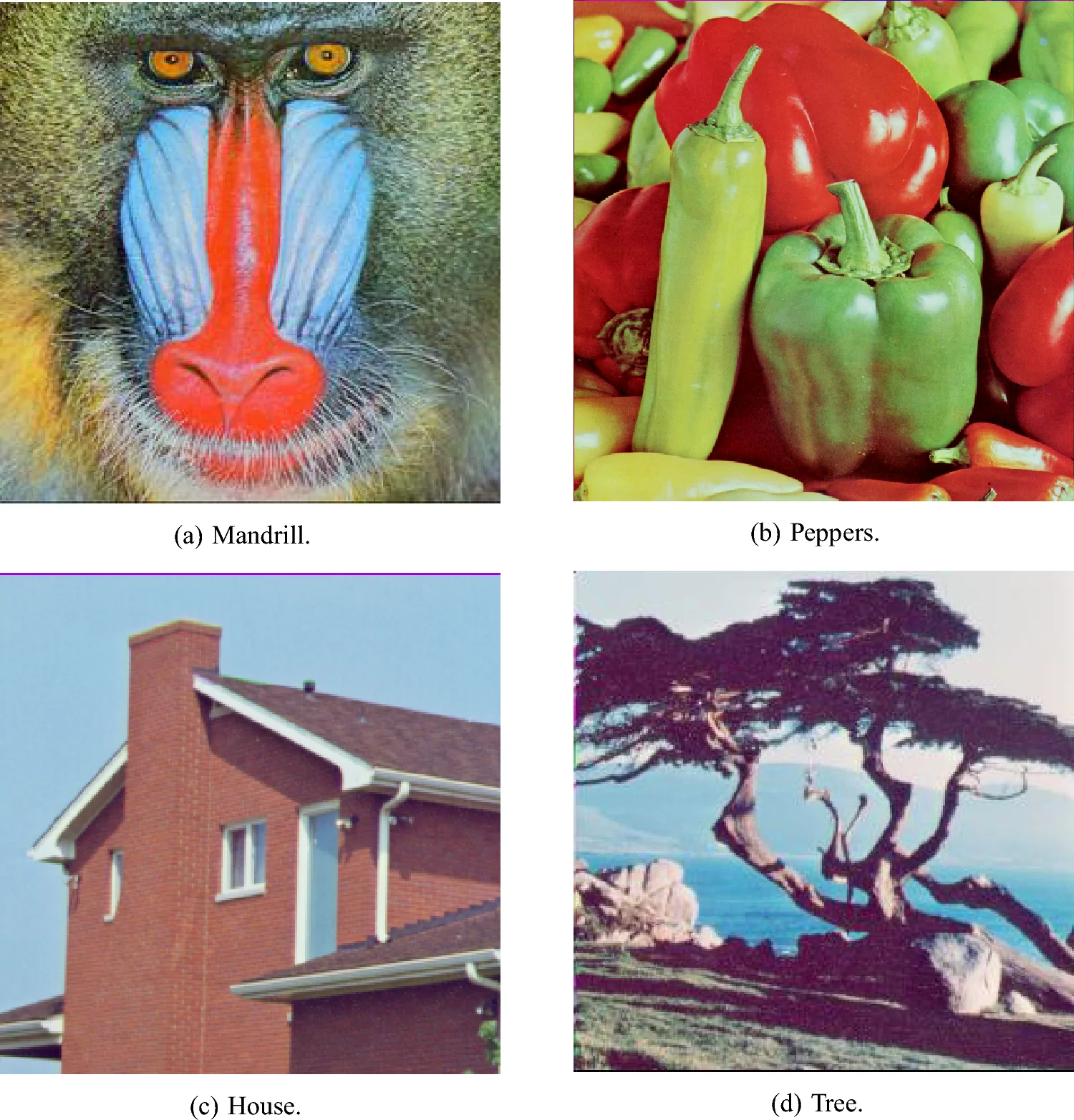
Plain images.

**Fig. 7:**
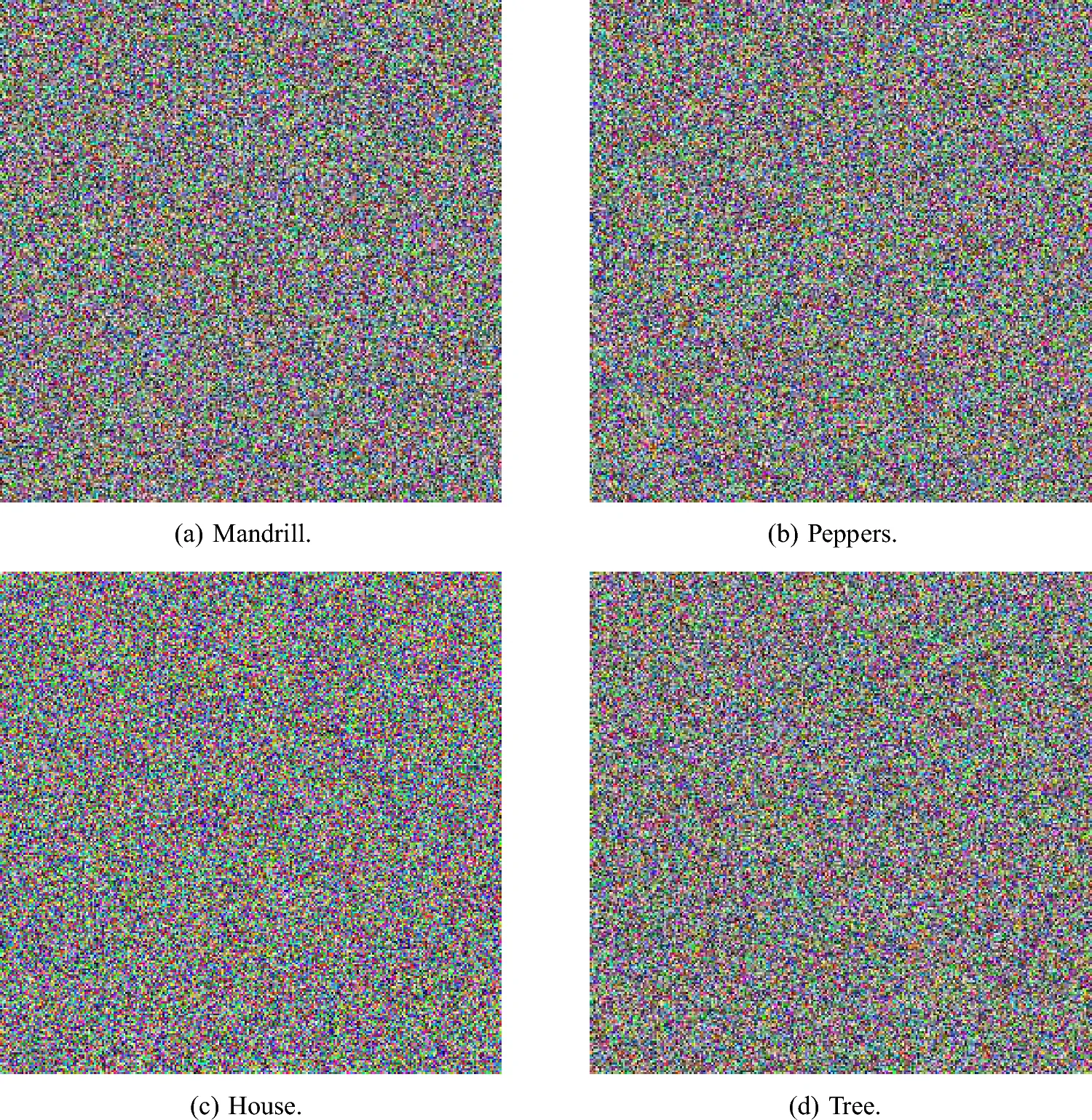
Encrypted images.

**Fig. 8:**
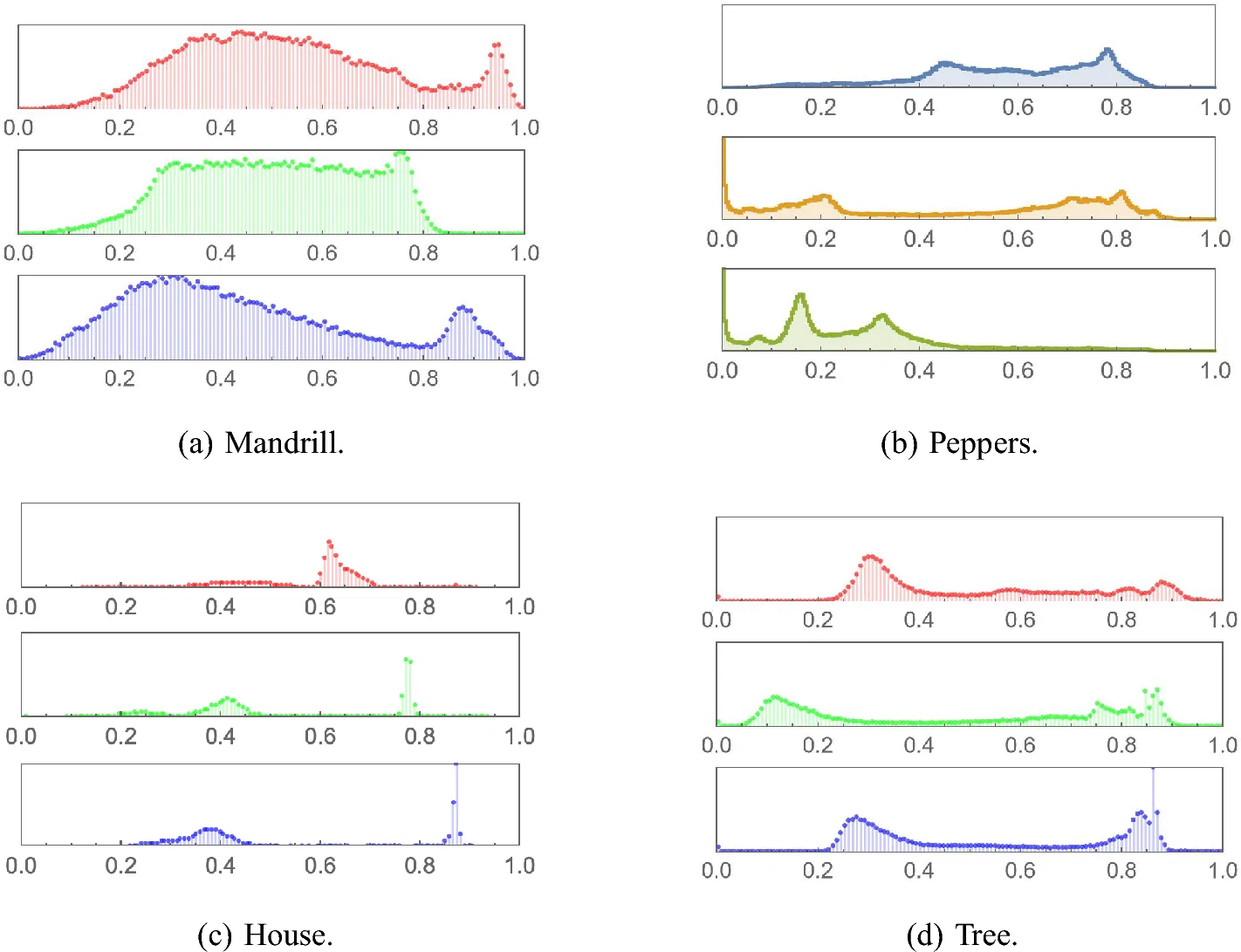
Plain images histograms.

**Fig. 9:**
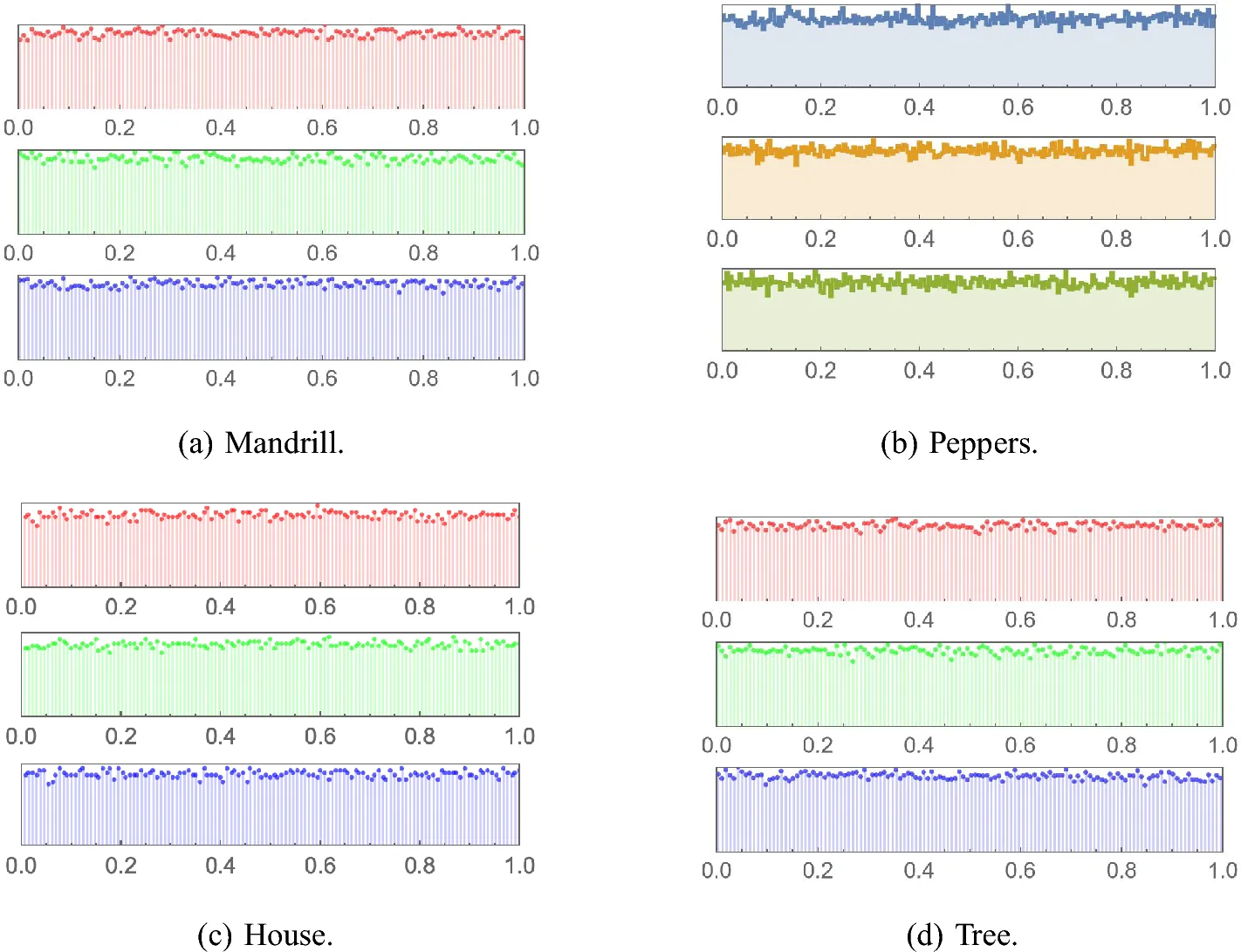
Encrypted images histograms.

Figures 6 and 7 display the plain and encrypted versions of different images. Also, Figs. 8 and 9 display their respective histogram plots. By comparing the histogram plots of the plain and encrypted versions of the images, one can observe any changes in the distribution of pixel values caused by the encryption process. Encryption algorithms are designed to randomize the pixel values in an image, so the histogram plot of the encrypted image shows a more uniform distribution of pixel intensities compared to the plain image.

**Fig. 10:**
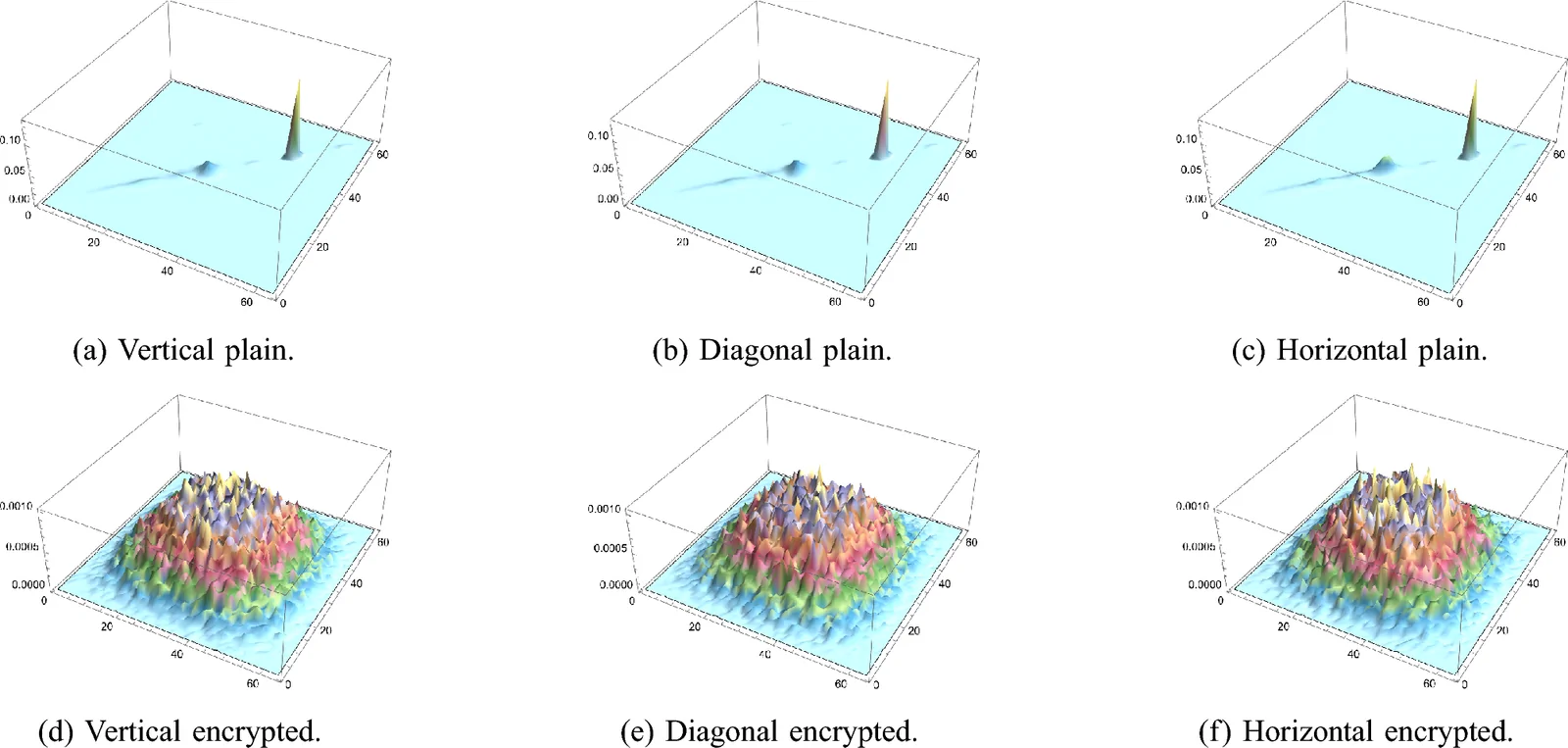
3D Correlation coefficient diagram of the plain and encrypted red channel of House image.

**Table 9:** Evaluation of the proposed scheme’s performance.

Image	MSE	PSNR [dB]	MAE	Entropy	NPCR [%]	UACI [%]
Peppers	10140.4	8.07024	82.3371	7.99894	99.5962	32.2891
Mandrill	8291.11	8.94468	75.0495	7.99893	99.6134	29.4312
Tree	9952.0	8.1517	81.5244	7.99914	99.6129	31.9704
House	8315.5	8.93192	75.0847	7.99895	99.6109	29.445
Average	9174.7525	8.52464	78.49893	7.99899	99.60835	30.78393

### Statistical analyses

The results presented in Table 9 demonstrate the strong performance of the proposed image encryption approach across multiple statistical metrics. The elevated MSE and reduced PSNR values confirm that the encryption process introduces substantial alterations, which is an essential characteristic for secure encryption. The MAE results further validate the presence of considerable pixel-level changes. Additionally, the entropy measurements reveal that the encrypted images possess high randomness approaching the ideal value of 8, reflecting strong confusion and diffusion properties. Overall, these metrics collectively affirm the robustness and reliability of the proposed algorithm in protecting image data.

Table 10 presents a comparison of the Pixel Correlation Coefficient (PCC) values for both plain and encrypted images in three directions: vertical (V), horizontal (H), and diagonal (D). The plain images exhibit high correlation values, with average PCCs of 0.91712400, 0.93759150 and 0.89062200 for the three orientations, reflecting the typical structural redundancy found in natural images. In contrast, the encrypted images show near-zero correlation, with average PCCs of *-0.00215014*, 0.00057989 and *-0.00287457* for the same directions. These results indicate that the proposed encryption method effectively disrupts pixel dependencies, introducing randomness. The analysis is further supported by the 3D PCC plots in Fig. 10, which display the correlation in all three directions for both the plain and encrypted versions of the Peppers image.

These findings validate the algorithm’s effectiveness in enhancing image security by removing the correlations typically found in plain images.

**Table 10:** Correlation coefficients of plain and encrypted images.

Plain image				Encrypted image		
Image	Vertical	Horizontal	Diagonal	Vertical	Horizontal	Diagonal
Peppers	0.96679500	0.95942200	0.93042600	-0.00279141	0.00194988	-0.00027927
Mandrill	0.79088000	0.84877800	0.75062400	-0.00482579	0.00153100	-0.00717397
Tree	0.94515000	0.96815300	0.92996700	-0.00595065	0.00028251	-0.00118117
House	0.952926	0.978232	0.936044	0.000875371	0.00111976	0.00546522
Average	0.91393775	0.93864625	0.88676525	-0.00317312	0.00122079	-0.00079230

###  Differential attack analysis

A way to evaluate the effectiveness of an encryption method against differential attacks is by using a differential attack analysis. This analysis can involve two metrics: NPCR and UACI. These methods are used to evaluate the encryption scheme’s ability to withstand differential attacks.

Table 9 presents estimated values for NPCR and UACI for several encrypted images, As shown, all achieved NPCR values are above *99% *, while the UACI is also around the value *33%*. As a result, any slight difference in the plaintext image would result in a significant difference in the encrypted image. The average NPCR and UACI values of the proposed scheme, as shown in Table 9, are *99.60897%* and *32.26675%*, respectively.

### Further attacks

The security of the proposed encryption scheme is also evaluated against additional classical cryptanalytic attack models. In the proposed method, the secret key material is generated from the synchronized final weight vectors through SHA-512, Mersenne Twister (MT), and Rule30CA, rather than being derived from the plaintext image itself. Moreover, the encryption process is carried out through multiple successive key-dependent stages, including XOR masking, dynamically generated S-box substitution, DNA encoding, DNA addition, and Rule30CA-based transformation. This multi-layer structure reduces the possibility of exploiting direct relationships between plaintexts and ciphertexts and enhances the robustness of the scheme against several attack scenarios.

#### Known-plaintext attack & known-ciphertext attack

**Known-Plaintext Attack (KPA):** In a known-plaintext attack, the adversary has access to one or more plaintext images and their corresponding ciphertexts and attempts to recover the secret key or infer the encryption procedure. The proposed scheme resists this attack because the keys $K_1$, $K_2$, $K_3$ are generated from the synchronized final weight vectors using SHA-512, MT, and Rule30CA, and thus remain independent of the plaintext image. In addition, the plaintext is processed through several key-dependent operations, including XOR with $K_1$, substitution using S-box 1, DNA addition with $K_{2DNA}$, XOR with $K_3$, and substitution using S-box 2. Therefore, knowledge of plaintext-ciphertext pairs alone does not provide sufficient information to reconstruct the secret parameters or recover the encryption keys.

**Known-Ciphertext Attack (KCA):** In a known-ciphertext attack, the attacker has access only to the encrypted images and attempts to recover the original plaintext without any prior knowledge of the secret keys. This attack remains ineffective against the proposed scheme because the ciphertext is produced by multiple nonlinear and key-controlled transformations applied in sequence. Without the secret key material required to generate $K_1$, $K_2$, $K_3$, S-box 1, and S-box 2, the attacker cannot correctly reverse the XOR stages, regenerate the substitution boxes, or invert the DNA addition process. Hence, ciphertext-only observations are insufficient to reveal the original image content.

#### Chosen-plaintext attack & chosen-ciphertext attack

*Chosen-plaintext attack (CPA):* In a chosen-plaintext attack, the adversary is allowed to select arbitrary input images and obtain their corresponding ciphertexts in an attempt to discover exploitable plaintext-ciphertext relations or derive the secret key. The proposed scheme shows resistance to this attack because the internal key-generation process is controlled by the synchronized final weight vectors and not by the chosen plaintext itself. More specifically, SHA-512, MT, and Rule30CA are used to generate the secret keys independently of the image content. Furthermore, the plaintext undergoes several successive key-dependent and nonlinear transformations, namely XOR masking, S-box substitution, DNA encoding and addition, and a final Rule30CA-based layer. As a result, even carefully designed plaintexts do not enable the attacker to isolate the secret parameters or recover the internal keys in a practical manner.

*Chosen-ciphertext attack (CCA):* In a chosen-ciphertext attack, the adversary attempts to decrypt selected ciphertexts in order to obtain useful information about the plaintext or the secret key. The proposed scheme is resistant to this attack because successful decryption requires the same secret parameters used during encryption to regenerate the correct keys and substitution boxes. In the absence of these parameters, arbitrary ciphertexts cannot be meaningfully reversed through the XOR, S-box, and DNA-based stages. Consequently, chosen-ciphertext queries do not provide practical information that can be used to recover the original plaintext or infer the secret key structure.

### Key sensitivity analysis

Key sensitivity is an important security requirement for image encryption schemes, where a very small change in the key should produce a completely different cipher image and should prevent successful decryption. In the proposed scheme, the encryption keys are derived from the final synchronized TPM weight vectors through SHA-512, and are then used to generate $K_1$, $K_2$, $K_3$, and the corresponding S-boxes. Therefore, to evaluate key sensitivity, one element of the final TPM weight vector is slightly modified while all other key parameters remain unchanged. Let the correct key be denoted by $\mathcal {K}$ and the slightly modified key by $\mathcal {K}'$, where

32 $$\begin{aligned} W'1(i,j)=W_1(i,j)+1. \end{aligned}$$

**Fig. 11:**
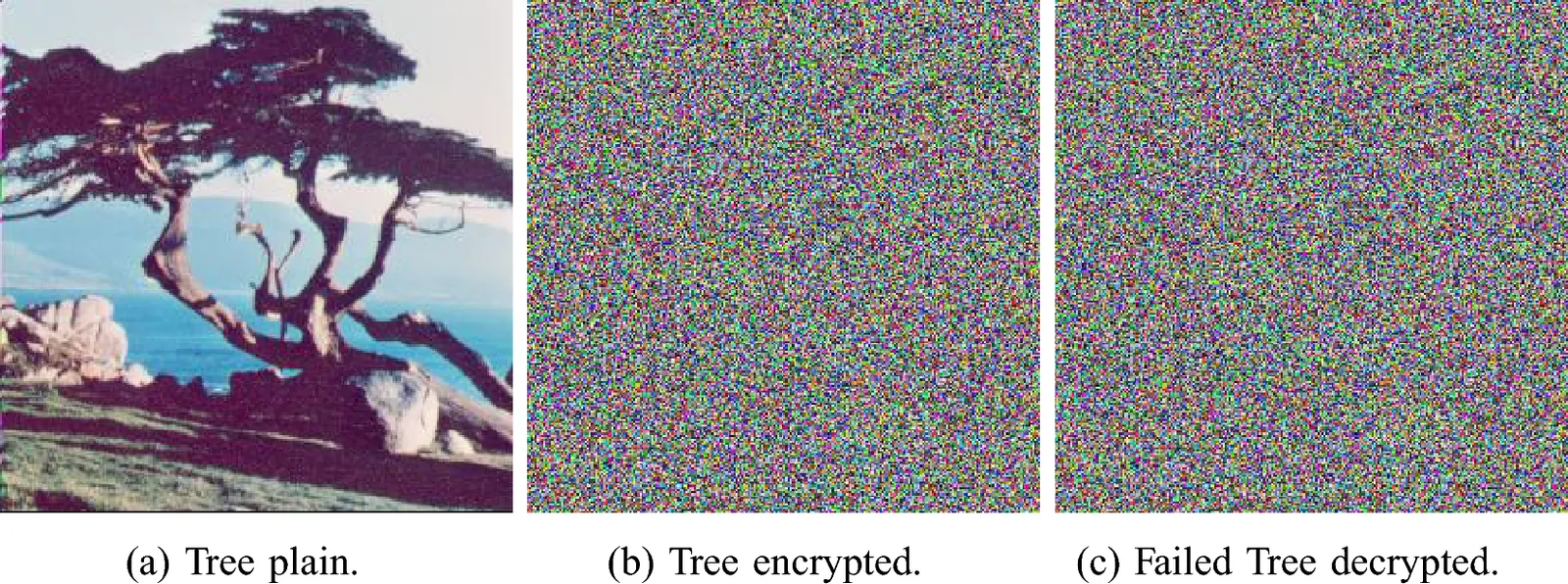
Key sensitivity visual analysis showing failed decryption using slightly changed key.

Decryption using $\mathcal {K}'$ fails to recover the original image and produces an unintelligible output, as shown in Fig. 11. This is because even a one-unit change in the TPM weight vector leads to a completely different SHA-512 output, which consequently changes the generated keys and S-boxes. Hence, the proposed scheme demonstrates strong sensitivity to the initial secret keys and is resistant to brute-force and key-guessing attacks.

### Execution time & computational complexity analyses

To determine the suitability of the proposed algorithm for real-time applications, an analysis of its encryption rate is conducted. The results are due to applying the encryption scheme on different images of dimensions *256 × 256*. As shown in Table 11 the proposed scheme takes 0.85846 s to encrypt an image. This translates to an encryption rate of 1.8 Mbps. The computational complexity of the proposed encryption scheme is linear with respect to the image size. Let the input image dimensions be *M × N*. The main operations in the three encryption layers, including bit-stream conversion, key-stream generation, XOR operations, DNA conversion, DNA addition, and S-box substitution, are all performed by scanning the image data once for a constant number of times. Therefore, the overall time complexity of the proposed scheme is *O(M× N)*.

**Table 11:** Execution time comparison for various schemes of the Lena image having dimensions *256× 256*.

Scheme	Encryption time [s]	Machine specifications	Computational
	[s]	(CPU and RAM)	Complexity
Proposed scheme	0.85846	2.7 GHz Intel^®^ Core^TM^ i7, 8 GB	*O(M× N)*
^25^	2.582389	2.9 GHz Intel^®^ Core^TM^ i9, 32 GB	*O(M× N)*
^45^	1.42545	2.9 GHz Intel^®^ Core^TM^ i9, 32 GB	*O(M× N)*
^46^	1.1168	3.4 GHz Intel^®^ Core^TM^ i7, 8 GB	*O(M× N)*

### Key space analysis

The key space of the proposed encryption scheme is determined by the number of possible values of its independent secret inputs. In the proposed method, the main secret material is represented by the TPM final weight vectors used to generate the seeds $S_1$ and $S_3$. In contrast, the internal values $K_1$, $K_2$, and $K_3$ are not counted as additional independent keys, since they are deterministically derived from the TPM secret material through SHA-512, MT, and Rule 30 CA.

According to Algorithm 1, each TPM weight $W_{i,j}$ takes an integer value from the set $\{-L,\ldots ,0,\ldots ,+L\}$. Hence, each weight has *(2L+1)* possible values. Since the TPM contains *N* hidden neurons and *K* input neurons connected to each hidden neuron, the total number of weights is *N × K*. Therefore, the number of possible TPM weight configurations corresponding to one synchronized final weight vector is given by $(2L+1)^{N\times K}$.

In the proposed encryption scheme, two TPM-derived final weight vectors, namely *finalWeightVector*1 and *finalWeightVector*2, are used to generate the seeds $S_1$ and $S_3$ for two different stages of the encryption process. Therefore, when these two TPM outputs are generated independently, the overall key space becomes $\left( (2L+1)^{N\times K}\right) ^2 = (2L+1)^{2NK}$.

For the parameter setting adopted in this work, *(N,K,L)=(100,10,10)*, the resulting key space is $(2\times 10 + 1)^{2\times 100\times 10} = 21^{2000} \approx 2^{8784}$.

This key space is sufficiently large to render exhaustive brute-force search computationally impractical. Therefore, the proposed scheme provides a high level of resistance against brute-force attacks (Table 12).

**Table 12:** Key space values comparison.

Scheme	Key space
Proposed scheme	$2^{8784}$
^24^	$2^{744}$
^25^	$2^{425}$
^45^	$2^{372}$
^47^	$2^{128}$
^48^	$2^{256}$
^49^	$2^{187}$

### Security of TPM-based key exchange

TPM synchronization is employed to establish a shared session secret over a public channel. Under a passive eavesdropping model, known TPM attacks (e.g., geometric/flipping-style attacks) may be attempted, in which synchronization of an attacker TPM is pursued using the observed input–output transcript. The effectiveness of these attacks is parameter-dependent and is influenced by the TPM structure (*K*, *N*, *L*) and the learning/update rule; therefore, (*K*, *N*, *L*) are explicitly treated as security parameters, and reliance is placed on established results in the TPM literature indicating that attacker convergence is significantly less likely and/or typically slower than legitimate synchronization for recommended parameter ranges.

Under an active adversary model, authentication is not provided by TPM synchronization alone, and vulnerability to MITM attacks is therefore implied, in which two independent synchronizations can be executed by the adversary and distinct keys can be established with each endpoint. To address this limitation, the key-establishment procedure must be combined with an authentication mechanism (e.g., MAC-based key confirmation or certificate/PSK-based authentication). This requirement is incorporated into the revised protocol description, and the corresponding security claims are updated accordingly.

###  The national institute of standards and technology analysis

Statistical randomness of the encrypted outputs is evaluated using the NIST SP 800-22 test suite, in which bit-streams derived from the encrypted images were tested against the randomness hypothesis at a typical significance level (e.g., *α =0.01*), and *p*-values and pass rates were reported. As shown in Table 13 (aggregated over the tested images, each represented by a bit-stream length over 1.5 MB), Pass outcomes were obtained for all listed tests, with pass rates largely reported as 1.000 and acceptable *p*-values (e.g., Frequency *p=0.487*, Runs *p=0.524*, Approximate Entropy *p=0.719*). A slight reduction is observed only for Random Excursions (Avg.) (pass rate 0.958), while an overall pass rate of 0.998 is maintained, indicating that strong pseudo-random behavior is achieved by the encrypted bit-streams.

**Table 13:** Detailed NIST suite results aggregated over the tested images.

Test name	Pass	*p*-	Result
	rate	value	
Frequency	1.000	0.487	Pass
Block Frequency	1.000	0.425	Pass
Cumulative Sums (Fwd.)	1.000	0.420	Pass
Cumulative Sums (Rev.)	1.000	0.464	Pass
Runs	1.000	0.524	Pass
Longest Run	1.000	0.681	Pass
Rank	1.000	0.264	Pass
FFT	1.000	0.619	Pass
Non-Overlapping Template (Avg.)	1.000	0.788	Pass
Overlapping Template	1.000	0.537	Pass
Universal (Maurer’s)	1.000	0.307	Pass
Approximate Entropy	1.000	0.719	Pass
Random Excursions (Avg.)	0.958	0.393	Pass
Random Excursions Variant (Avg.)	1.000	0.514	Pass
Serial 1	1.000	0.607	Pass
Serial 2	1.000	0.593	Pass
Linear Complexity	1.000	0.455	Pass
Overall	0.998	—	Pass

### Comparative analysis with the literature

In this section, Table 14 presents a comparative analysis of the performance of the proposed scheme with various image encryption algorithms. Notably, the proposed algorithm demonstrates a low PSNR and a high MAE relative to most alternative methods, which is a positive outcome in this context. A reduced PSNR indicates that the encrypted image bears less resemblance to the original, while a higher MAE signals more substantial alterations to the image. These characteristics are advantageous, as they imply stronger encryption. The entropy values are nearly at their maximum, and the very low or even negative correlation coefficients further validate the randomness of the encryption process, ensuring minimal similarity to the original image. These factors make the encryption more resilient to statistical analysis and attacks. Furthermore, the algorithm’s high NPCR and reasonable UACI values validate the significant sensitivity and intensity of the modifications, reinforcing the algorithm’s ability to secure sensitive geographic data effectively. Moreover, the results displayed in Table 11, demonstrate that the encryption rate of the proposed scheme is faster than that of all the compared algorithms. The encryption rate of an encryption scheme is influenced by several factors, including the algorithm itself, machine specifications, and software configuration.

In summary, these comparative results demonstrate that the proposed encryption method is highly suited for high-security applications where maintaining data integrity and confidentiality is critical (Table 15).

**Table 14:** Comparative metrics overview.

Image	Scheme	MSE	PSNR	MAE	Entropy	CC			NPCR	UACI
						V	H	D		
Peppers	Proposed	10140.4	8.07024	82.3371	7.99894	-0.00279141	0.00194988	-0.000279271	99.5962	32.2891
	^24^	10049.4	8.10939	81.8995	7.99716	-0.0015191	-0.0020878	0.00065688	-	-
	^25^	10092.3	8.09089	81.7740	7.99877	-0.00102	-0.00063	-0.00003	99.59360	32.17523
	^26^	-	8.09689	82.0274	7.99908	0.00580309	0.00245556	-0.00265354	99.6272	32.1676
	^27^	10897.50	7.7577	92	7.9998	0.0008	0.0013	0.0011	-	-
	^28^	-	8.1156	-	7.9991	-	-	-	-	-
	^29^	7274.44	9.55	-	-	-0.0028	-0.0090	-0.0007	99.5626	33.5486
	^45^	10298.7	8.00296	82.3273	7.9951	0.001043	0.004331	0.001856	99.6348	32.1832
	^46^	-	31.2463	-	7.5918	-0.00279141	0.00194988	-0.000279271	99.6155	33.5595
Mandrill	Proposed	8291.11	8.94468	75.0495	7.99893	-0.00482579	0.001531	-0.00717397	99.6134	29.4312
	^24^	8333.14	8.92272	75.3317	7.99743	-0.0019799	0.0013408	-0.004713	-	-
	^25^	8295.21	8.94253	75.1659	7.99907	-0.00138	0.001362	-0.00332	99.58190	29.39764
	^26^	-	8.93946	75.1327	7.99914	-0.00298955	-0.00309558	-0.00007469	99.6185	29.4638
	^27^	10930.33	7.7447	92	7.9991	0.0011	-0.0001	0.0003	99.61	33.51
	^28^	-	8.9695	-	7.9991	-	-	-	-	-
	^29^	6399.05	10.10	-	-	-0.0029	-0.0079	0.0026	99.5926	33.4496
	^45^	8573.38	8.79929	81.913	7.9905	0.003522	-0.00484	0.003429	99.6124	29.3973

**Table 15:** Evaluation of the proposed S-boxes.

S-box	NL	SAC	BIC	LAP	DAP
$SBox_{\text {MT}}$	108	0.501709	108	0.078125	0.015625
$SBox_{\text {CA}}$	108	0.500244	108	0.078125	0.015625
Ideal values	112	0.5	112	0.0625	0.015625

## Conclusions

This article presented a 3-level scheme to image encryption. The scheme combines TPMs for secure key exchange, DNA coding, MT-based DNA sequences generation, SHA-512 seeded Rule 30 Cellular Automata for generating a PRNG, and S-box construction for ultimate encryption. Each technique offers unique advantages and employs distinct mathematical principles to achieve encryption. The proposed scheme’s performance is evaluated through a variety of tests and measurements, including histogram, pixel cross-correlation, entropy, MSE, PSNR calculations, and others that demonstrated excellent results in terms of security, resistance to attacks, and computational efficiency. Upon comparing it with counterpart algorithms from the literature, its security was found to be highly comparable.

### Limitations

Despite being designed for efficient cloud-based image exchange, the proposed scheme has some limitations. (i) Computational complexity: the multi-stage pipeline (bit-stream conversion, XOR, S-box substitution, DNA operations, and CA-based masking) adds cumulative cost that grows with image size, and Algorithm 3 may incur extra overhead depending on the threshold and iterations needed to accept an S-box. (ii) Synchronization overhead: TPM key establishment is interactive and may introduce latency and communication overhead; decryption also requires strict agreement on all parameters (TPM settings, seeds, thresholds, and mapping rules). (iii) Scalability: high-resolution images, many concurrent sessions, or frequent key refresh can stress compute/network resources due to per-session TPM synchronization and memory-intensive transforms, motivating optimized implementations and larger-scale cloud benchmarking.

### Future work

Further research could include: (i) investigate the feasibility of combining the proposed algorithm with homomorphic encryption techniques to enable secure computation on encrypted data^50^, (ii) carry out a hardware implementation for improved encryption throughput^51^, and (iii) explore the utilization of other CA rules^52^, as well as higher order CA^53^.

## Data Availability

The datasets used and/or analyzed during the current study are available from the corresponding author on reasonable request.
